# Strategy for modular assembly of tetravalent, multispecific antibodies

**DOI:** 10.1002/pro.70797

**Published:** 2026-09-18

**Authors:** Evan Mallette, Levi L. Blazer, Craig A. Hokanson, Chao Chen, Julia Garcia Perez, Alevtina Pavlenco, Lynda Ploder, Alexander U. Singer, Michael D. L. Suits, Sunil Bhakta, Jagath R. Junutula, Jarrett J. Adams, Sachdev S. Sidhu

**Affiliations:** ^1^ The Anvil Institute of Systems Biologics Toronto Ontario Canada; ^2^ Department of Chemistry and Biochemistry Wilfrid Laurier University Waterloo Ontario Canada; ^3^ Aarvik Therapeutics Hayward California USA; ^4^ Simisco Biosciences Hayward California USA

**Keywords:** antibody, CD30, drug design, inhibitor, multispecific antibody, multivalent antibody, PD‐L1, phage display, protein engineering, structural biology

## Abstract

Multispecific, multivalent antibodies (Abs) are a burgeoning class of drugs that dramatically expand the pharmacological repertoire beyond traditional therapeutic Abs. Here, we present a simple, modular approach to developing multispecific, multivalent Abs based on a Fab‐phage library with a single light chain. Using this library, we created three Abs targeting unique sites on programmed death‐ligand 1 (PD‐L1) and another antibody targeting CD30. Biophysical and cellular characterization of these Abs demonstrated their functional equivalence to clinically relevant Abs targeting PD‐L1 or CD30. We then combined these paratopes into a series of bispecific, tetravalent, triparatopic Abs that retained the functionality of the parental Abs. Structural analysis of each of the Abs in complex with their cognate antigens demonstrated the adaptability of the common light chain to form diverse paratopes with an array of distinct heavy chains.

## 1 INTRODUCTION

**FIGURE 1:**
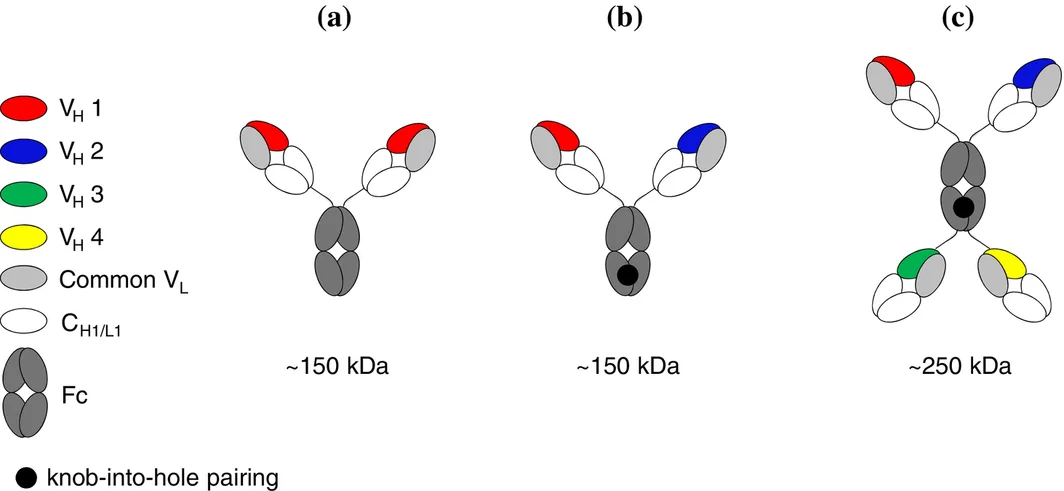
Antibody formats. Schematics are shown for (a) bivalent, monospecific immunoglobulin G (IgG), (b) bivalent, bispecific IgG (bslgG), and (c) tetravalent, tetraspecific Fab‐Fc*‐Fab. Fab, antigen‐binding fragment; Fc, central fragment crystallizable.

Over the past quarter century, monoclonal antibodies (Abs) have become the dominant class of biologics for treatment of numerous diseases, including cancer, autoimmune, and infectious diseases. Abs possess many advantages for drug development, including high specificity and potency, low immunogenicity, facile manufacturing, and the ability to target virtually any extracellular protein of interest. Currently, there are over 200 approved therapeutic Abs worldwide (Lyu et al., 2022). Most therapeutic Abs approved to date have been built in the natural bivalent immunoglobulin G (IgG) format, which consists of two identical antigen‐binding fragments (Fabs) linked to a central fragment crystallizable (Fc) domain that mediates effector functions (Figure 1a). However, despite the considerable success of therapeutic IgGs, the ability to target only one epitope with two identical binding sites limits many therapeutic applications that could be achieved with higher valency and/or expanded epitope targeting.

Consequently, considerable effort has been expended on developing bispecific Ab formats that enable targeting of two distinct epitopes, either on the same target or on different targets. The earliest and most validated bispecific format is the bivalent, bispecific IgG that relies on a heterodimeric “knob‐into‐hole” Fc (Fc*) (Ridgway et al., 1996) to assemble two distinct Fab arms with different heavy chains and either a common light chain (Sidhu et al., 2004) or light chains otherwise designed to pair with a specific heavy chain (Figure 1b). Many Abs of this type have been successfully manufactured and moved into the clinic, and several have been approved for therapy, thus validating bispecific IgGs as a novel drug class that can further expand the scope of therapeutic Abs (Klein et al., 2024; Shan et al., 2025). Interestingly, nature has also adopted the concept of bispecific IgGs for specialized Ab functions. In particular, the IgG4 subclass of Abs can naturally undergo Fab arm exchange to form native bispecific molecules that possess unique immunological properties (Rispens & Huijbers, 2023; van der Neut Kolfschoten et al., 2007).

In parallel, other work has focused on expanding the valency of Abs by appending additional binding arms to an IgG to generate tetravalent Abs targeting two or more distinct epitopes (Herrera et al., 2024). To circumvent the need for a common light chain, as would be the case with Fabs, the additional arms are commonly derived from non‐natural peptides, cytokines, and other natural proteins, autonomous V_H_H domains that lack a light chain, or single‐chain variable fragments and diabodies in which the heavy and light chains are directly linked. Recently, we have combined the knob‐into‐hole and diabody technologies to assemble a tetravalent, trispecific format that proved critical for efficient activation of the Wnt/Fzd signaling complex, which requires receptor clustering beyond dimerization (Chidiac et al., 2021; Tao et al., 2019). Although these structurally diverse additional arms have broadened the utility of Ab therapeutics, most suffer from reduced stability compared with Fabs, and their non‐natural fusions to natural Abs raise concerns of immunogenicity. Thus, it would be ideal to engineer multiparatopic Abs with arms composed only of Fabs.

Here, we report the development of a Fab‐Fc*‐Fab format (Figure 1c) and a strategy that permits modular assembly of tetraparatopic Abs consisting of a central Fc* heterodimer with two different Fabs fused to the N‐termini and two other different Fabs fused to the C‐termini. To achieve modularity, we designed a synthetic phage‐displayed Fab library consisting of diversified heavy chains together with the light chain of Trastuzumab (TRAS), a clinically approved IgG that targets the receptor tyrosine kinase HER2/ErbB2. We used these libraries to generate three Fabs targeting the immune checkpoint receptor PD‐L1 and one Fab targeting the classical Hodgkin's Lymphoma (cHL) marker CD30 (van der Weyden et al., 2017). In order to understand how a common light chain adapts to each unique paratope, we performed structural analysis of the interaction between the individual Fabs and their cognate targets. These crystal structures revealed how the unique heavy chain of each Fab mediates high affinity with specificity, and how the common light chain adapts to support diverse molecular interactions.

To demonstrate the utility of our modular assembly approach, we constructed triparatopic Fab‐Fc*‐Fab Abs targeting two epitopes on PD‐L1 with two different arms and a single epitope on CD30 with two identical arms. We showed that these Abs can activate T cells via their anti‐PD‐L1 arms, and they internalize efficiently into cancer cells displaying CD30. Multiparatopic Abs of this type may be promising therapeutics for treating cancers that depend on several signaling pathways, and they may also enhance specific targeting of tumors. Moreover, this general strategy provides a means for assembling Fab building blocks into multifunctional biologics with potential therapeutic applications for numerous diseases that remain recalcitrant to conventional IgG drugs.

## 2 RESULTS AND DISCUSSION

### 2.1 Construction and validation of a phage‐displayed fab library with a fixed light chain

**FIGURE 2:**
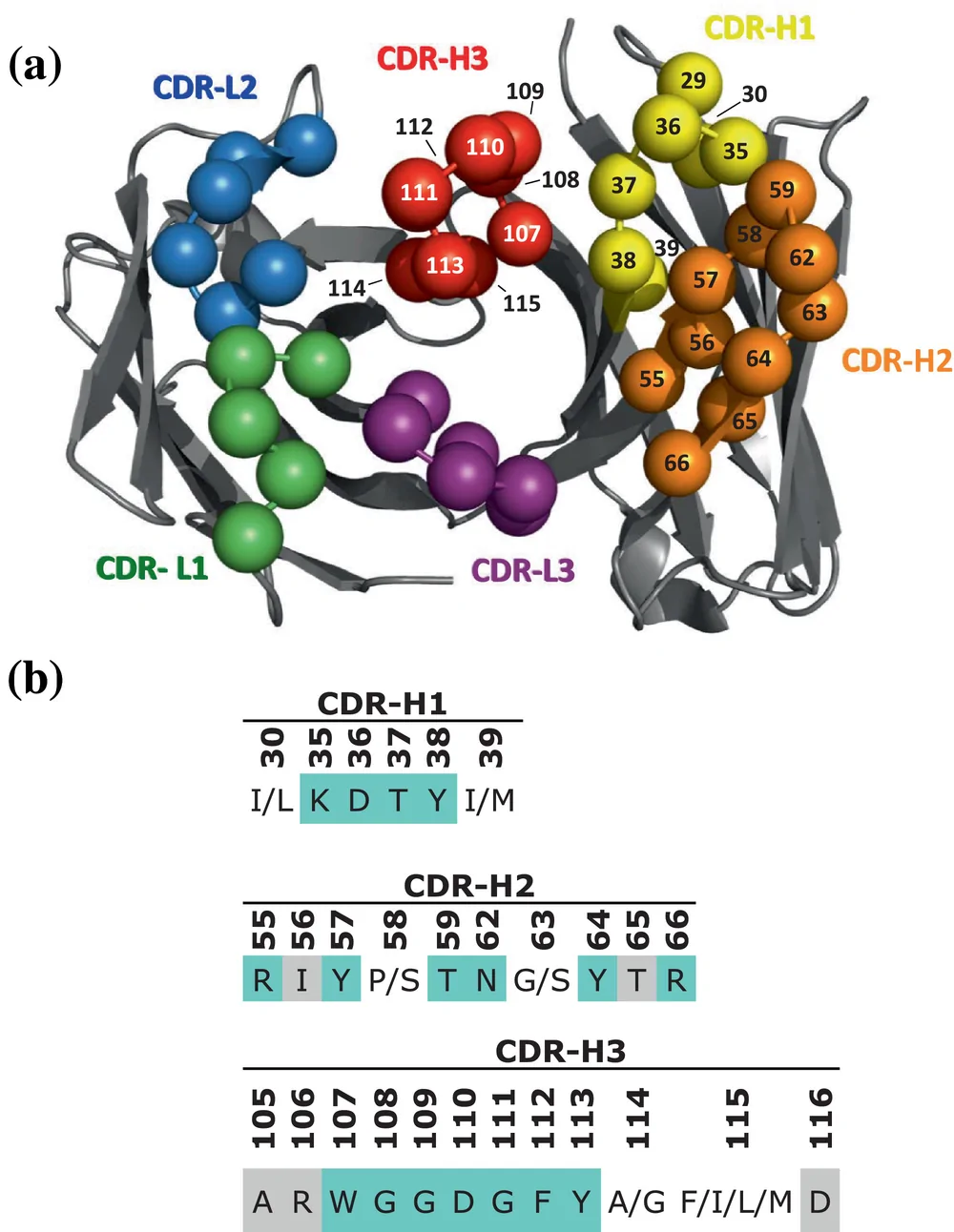
Fixed light‐chain Fab library design. (a) The structure of the paratope with complementarity determining region (CDR) positions shown as spheres colored as follows: H1 (yellow), H2 (orange), H3 (red), L1 (green), L2 (blue), L3 (purple). Heavy‐chain positions that were diversified are numbered according to the International Immunogenetics Information System (IMGT) scheme (Lefranc et al., 2003). The structural image was generated from Protein Databank (PDB) entry 1FVC. (b) The sequences of the heavy‐chain CDRs for the template used for library construction. Positions shaded in gray were fixed. Positions shaded in cyan were subjected to “soft randomization” with degenerate codons that allowed 50% wild type amino acid and 50% random mutation. Other positions were diversified by binary or tetranomial codons that encoded for the listed amino acids.

We previously developed a highly functional synthetic phage‐displayed Fab library (library F) built using the same framework as TRAS (Persson et al., 2013). Library F contains tailored synthetic diversity in the three heavy‐chain complementarity determining regions (CDRs) and in the third light‐chain CDR, and it has been used to generate thousands of Fabs targeting hundreds of diverse antigens. However, the use of a diversified light chain precludes modular assembly of multispecific Abs, so we designed and constructed a new library with the light chain sequence fixed as that of TRAS and diversity incorporated into the heavy‐chain CDRs (Figure 2). For most residues in this library, a “soft randomization” strategy was used to incorporate ~50% random substitutions at diversified positions within the TRAS background sequence. To maintain paratope stability, certain positions in the CDRs were either fixed or limited in their diversity. This library contained 5.0 × 10^9^ unique clones and phage representing the library were pooled and used in binding selections to isolate Fabs specific for PD‐L1 or CD30.

### 2.2 Development of anti‐PD‐L1 Abs

**FIGURE 3:**
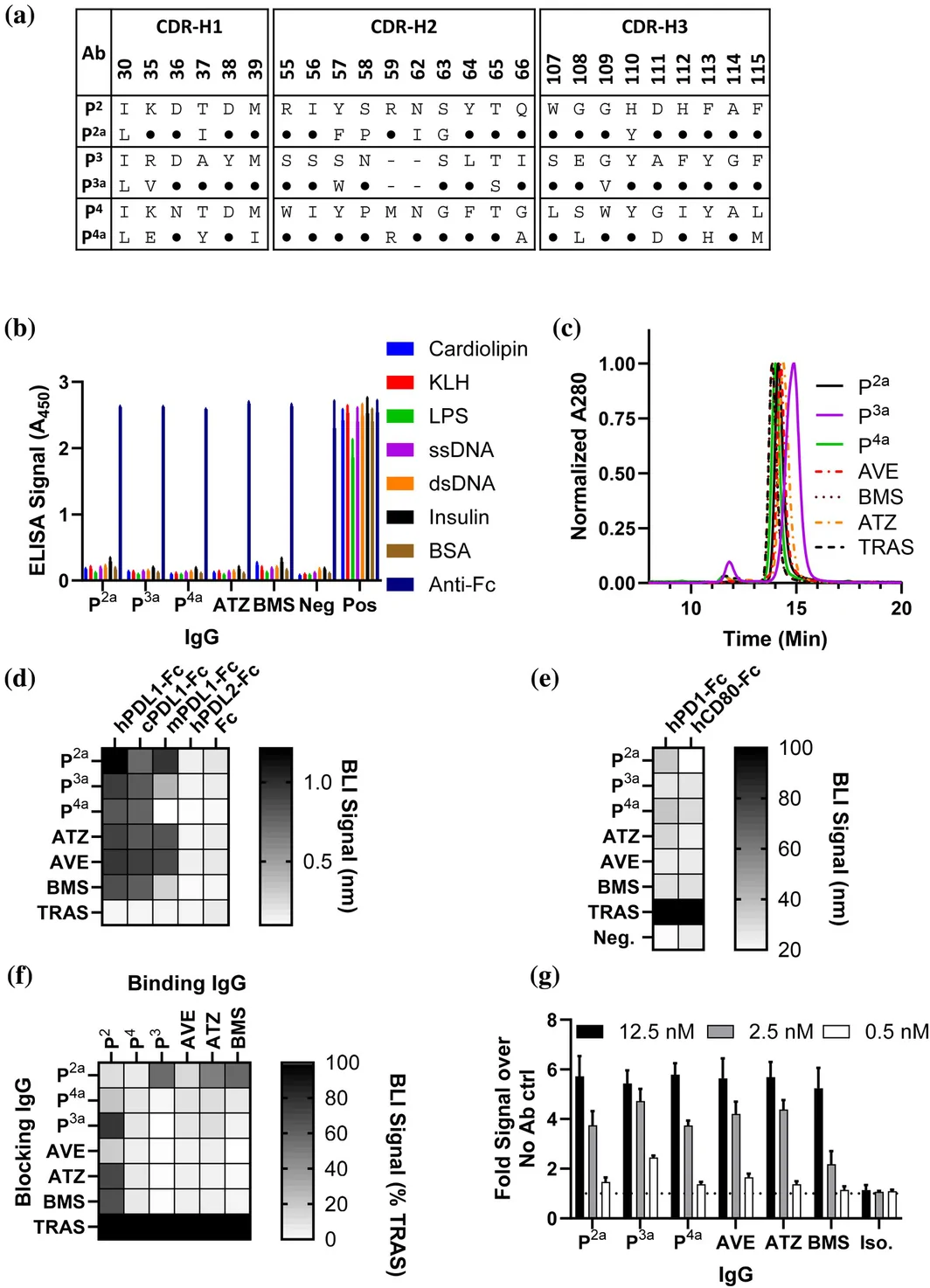
Characterization of anti‐PD‐L1 antibodies (Abs). (a) Heavy‐chain complementarity determining region (CDR) sequences of parental Abs (P^2^, P^3^, and P^4^) and their optimized derivatives (P^2a^, P^3a^, and P^4a^). Dots denote identities and dashes denote gaps in the alignment. (b) Polyreactivity enzyme‐linked immunosorbent assay (ELISA) to detect non‐specific binding to a panel of heterologous biomolecules (*y*‐axis) for IgGs P^2a^, P^3a^, and P^4a^, anti‐PD‐L1 IgGs Atezolizumab (ATZ) and BMS‐936559 (BMS), and negative control (Neg) and positive control (Pos) Abs that do not or do exhibit non‐specific binding, respectively (*x*‐axis). Abs were tested at 100 nM. Data are presented as the average of two to three independent experiments performed in duplicate. (c) Analytical gel filtration size exclusion chromatography of IgGs P^2a^ (black), P^3a^ (purple), and P^4a^ (green) and clinically approved IgGs Avelumab (AVE) (dashed red), BMS (dashed purple), ATZ (dashed orange), and TRAS (dashed black). Representative data from three runs are shown. (d) Biolayer interferometry (BLI) assays for binding of IgGs (vertical) to the indicated immobilized antigens (horizontal). (e) BLI competition assay for binding of human PD‐1 or CD80 extracellular domains (ECDs) (horizontal) to immobilized human PD‐L1 ECD saturated with the indicated IgGs (vertical). (f) BLI epitope conservation assay. Binding of the indicated IgGs (horizontal) to immobilized human PD‐L1 ECD was measured in the presence of the indicated blocking IgGs (vertical). Binding signals are expressed as percentage of Trastuzumab (TRAS) signal, which is a non‐competing control. (g) PD‐L1 signaling cell‐based reporter assay. Jurkat T cells stably expressing human PD‐1 and an NFAT‐driven luciferase reporter were co‐cultured with a PD‐L1‐expressing CHO‐K1 cell line. The cultures were treated with IgGs (*x*‐axis) at the indicated concentrations and luciferase activity was measured by luminescence and normalized to signal without IgG treatment (*y*‐axis). Inhibition of PD‐L1 signaling results in an increase in luciferase activity. Iso., Isotype control IgG.

The selection for anti‐PD‐L1 Fabs yielded three unique clones (P^2^, P^3^, and P^4^) (Figure 3a). Notably, one of the three Abs (P^3^) contains a shortened CDR‐H2 that does not conform to the library design and is likely a by‐product of the mutagenesis reaction used to create the library. To develop derivatives of each Fab with higher affinities, we constructed phage‐displayed sub‐libraries using a soft randomization process whereby positions in the heavy‐chain CDRs were allowed to be approximately 50% parental and 50% diversified. Paratopes that resulted from selecting the sub‐libraries for binding to PD‐L1 were produced in the bivalent IgG format via transient transfection in mammalian cells and screened for affinity using biolayer interferometry (BLI). For each parental Ab, we identified optimized variants (P^2a^, P^3a^, and P^4a^; Figure 3a) that were used in subsequent studies. Production of these Abs and affinity purification with Protein‐A chromatography yielded homogenous proteins, as determined by sodium dodecyl sulfate polyacrylamide gel electrophoresis (SDS‐PAGE) analysis (Figure S1a), with expression titers of 450, 220, and 180 mg of protein per liter of culture supernatant for P^2a^, P^3a^, and P^4a^, respectively. In a polyreactivity enzyme‐linked immunosorbent assay (ELISA), none of the optimized IgGs exhibited significant binding to a panel of common macromolecules (Figure 3b), a property that has been shown to predict good developability for therapeutic Abs (Jain et al., 2017; Mouquet et al., 2010). Analytical size exclusion chromatography (SEC) showed that all three IgGs eluted predominantly as monodisperse peaks with elution times similar to those of TRAS and three clinical anti‐PD‐L1 IgGs (Avelumab [AVE], Atezolizumab [ATZ], and BMS936559 [BMS]), another property indicative of good developability (Figure 3c).

Analysis of binding kinetics by BLI demonstrated that the IgGs bound to human PD‐L1 with high affinities (*K*_D_ = 1.0–3.1 nM) comparable to those of the three clinical IgGs (*K*_D_ = 1.0–1.9 nM; Table 1). BLI assays also showed that IgGs P^2a^ and P^3a^ bound to mouse PD‐L1 (mPD‐L1) and cynomolgus monkey PD‐L1 (cPD‐L1), whereas IgG P^4a^ bound to cPD‐L1 but not to mPD‐L1 (Figure 3d). None of the molecules bound to the closely related human PD‐L2 (Figure 3d). Moreover, competitive BLI assays demonstrated that all IgGs blocked binding of PD‐L1 to the natural ligands PD‐1 and CD80 (Figure 3e). Epitope binning experiments were performed by first blocking immobilized PD‐L1 with one IgG and then assessing binding of a second IgG (Figure 3f). As expected, each optimized IgG was blocked by its parental IgG, but differed in binding patterns with other blocking IgGs. IgG P^2a^ was blocked by IgG P^4^ and AVE, but not by IgG P^3^, ATZ, and BMS. IgG P^3a^ was blocked by all other IgGs except P^2^, and IgG P^4a^ was blocked by all other IgGs. Most importantly, we tested these Abs for their ability to inhibit PD‐1/PD‐L1 signaling using a commercially available assay in which PD‐1‐expressing Jurkat T cells harboring a luciferase reporter are co‐cultured with engineered PD‐L1‐expressing CHO‐K1 cells. In this assay, interaction of PD‐1 with PD‐L1 prevents reporter expression, while inhibition of the interaction by an Ab activates the T cells, resulting in expression of luciferase. All three anti‐PD‐L1 IgGs activated human T cells with activities comparable to those of the clinical IgGs (Figure 3g). Collectively, these studies showed that the three IgGs bind with high affinity to PD‐L1 in a manner that functionally blocks the PD‐1/PD‐L1 interaction and signaling. Moreover, cross‐blocking experiments demonstrated that the IgGs target different epitopes on PD‐L1, and in particular, that IgGs P^2a^ and P^3a^ can bind simultaneously to PD‐L1.

**TABLE 1:** Kinetics for antibodies binding to immobilized human antigens, determined by biolayer interferometry.

Ab	Antigen	*k*_on_ (1/Ms)	*k*_off_ (1/s)	*K*_D_ (nM)
P^2a^	PD‐L1	2.8 × 10^5^	2.9 × 10^−4^	1.0
P^3a^	PD‐L1	4.5 × 10^5^	8.9 × 10^−4^	2.0
P^4a^	PD‐L1	7.2 × 10^5^	2.2 × 10^−3^	3.1
AVE	PD‐L1	4.2 × 10^5^	4.1 × 10^−4^	1.0
ATZ	PD‐L1	7.6 × 10^5^	1.1 × 10^−3^	1.5
BMS	PD‐L1	8.7 × 10^5^	1.6 × 10^−3^	1.9
C^1^	CD30	4.6 × 10^5^	1.3 × 10^−3^	2.8
BRNT	CD30	3.7 × 10^5^	1.3 × 10^−3^	3.4
AV‐P087	PD‐L1	3.1 × 10^4^	4.2 × 10^−4^	25
AV‐P087	CD30	1.4 × 10^6^	1.3 × 10^−3^	0.91
AV‐P088	PD‐L1	1.8 × 10^4^	6.8 × 10^−4^	51
AV‐P088	CD30	1.4 × 10^6^	1.3 × 10^−3^	0.90
AV‐P089	PD‐L1	2.4 × 10^4^	4.8 × 10^−4^	22
AV‐P089	CD30	1.0 × 10^6^	1.3 × 10^−3^	1.3
AV‐P090	PD‐L1	5.6 × 10^4^	6.3 × 10^−4^	12
AV‐P090	CD30	1.1 × 10^6^	1.3 × 10^−3^	1.2

Abbreviations: Abs, antibodies; ATZ, Atezolizumab; AVE, Avelumab; BMS, BMS936559; BRNT, Brentuximab.

### 2.3 Development of an anti‐CD30 Ab

**FIGURE 4:**
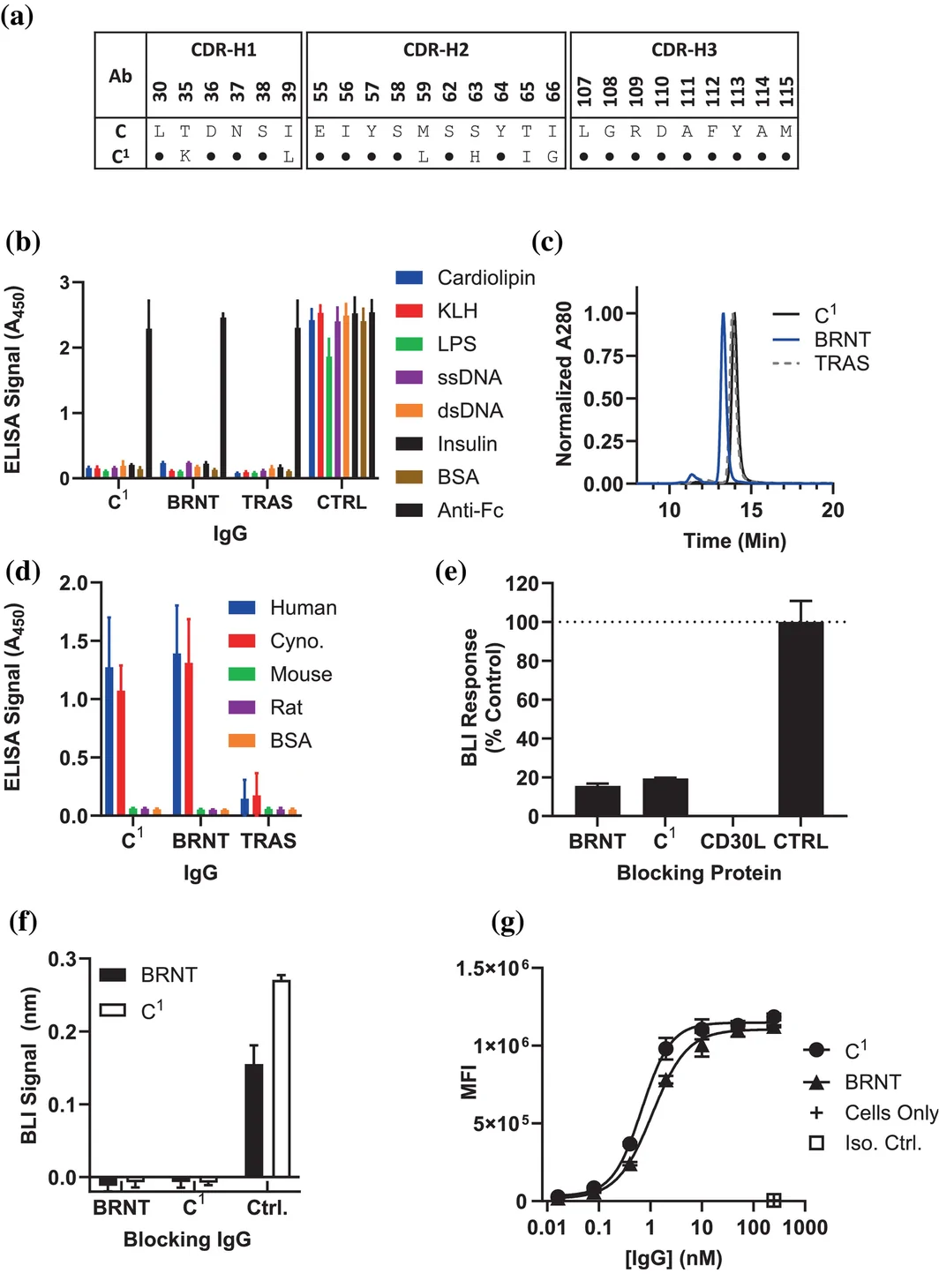
Characterization of anti‐CD30 Abs. (a) Heavy‐chain complementarity determining region (CDR) sequences of C and C^1^ antibodies (Abs). Dots denote identities. (b) Polyreactivity enzyme‐linked immunosorbent assay (ELISA) to detect non‐specific binding to a panel of heterologous biomolecules (*y*‐axis) for IgG C^1^, Brentuximab (BRNT), Trastuzumab (TRAS), and a positive control Ab (CTRL) that exhibits non‐specific binding (*x*‐axis). Abs were tested at 100 nM. Data are presented as the average of two to three independent experiments performed in duplicate. (c) Analytical gel filtration size exclusion chromatography of IgGs C^1^ (black), BRNT (blue), and TRAS (dashed black). Representative data from three runs are shown. (d) ELISA for IgGs (*x*‐axis) binding to immobilized CD30 from human, cynomolgus monkey, mouse, and rat (*y*‐axis). (e) Biolayer interferometry (BLI) competition assay for CD30L extracellular domain (ECD) binding to immobilized CD30 ECD (*y*‐axis) saturated with the indicated blocking protein (*x*‐axis). The BLI response (*y*‐axis) was normalized to the response for the negative control blocking protein. (f) BLI epitope binning assay. Binding of IgG C^1^ (white) or BRNT (black) to immobilized CD30 ECD was measured in the presence of the indicated blocking IgG (*x*‐axis). The plotted BLI signal (*y*‐axis) was corrected for baseline binding. (g) Flow cytometry analysis of serial dilutions of indicated IgGs (*x*‐axis) binding to L540 cells (*y*‐axis).

We also used the fixed light chain library to derive Abs targeting human CD30. As performed for PD‐L1, we identified an initial paratope (C, Figure 4a) from the naive library, then optimized it using a phage‐displayed sub‐library in which we diversified the heavy‐chain CDRs of the parental clone. From this sub‐library, we identified paratope C^1^ (Figure 4a), expressed it as an IgG in mammalian cells, and purified it in high yield by Protein‐A affinity chromatography (250 mg of protein per liter of cell culture). IgG C^1^ was highly pure by SDS‐PAGE (Figure S1b) and exhibited very low binding in the polyreactivity ELISA (Figure 4b). By analytical SEC, IgG C^1^ eluted as a monodisperse peak with an elution time similar to that of TRAS (Figure 4c).

Analysis of binding kinetics by BLI showed that IgG C^1^ bound to human CD30 with high affinity, comparable to that of the clinically approved Ab Brentuximab (BRNT; *K*_D_ = 2.8 or 3.4 nM, respectively; Table 1). ELISAs with immobilized antigens showed that IgG C^1^ bound to human and cynomolgus monkey CD30 but not to mouse or rat CD30 (Figure 4d). BLI assays also showed that both IgG C^1^ and BRNT blocked binding of CD30 to its ligand CD30L (Figure 4e) and they blocked each other from binding to CD30 (Figure 4f). Finally, flow cytometry assays with the Hodgkin's Lymphoma cell line L540 showed that IgG C^1^ recognized endogenously expressed cell‐surface CD30 as effectively as BRNT (Figure 4g, EC_50_ = 1.6 or 2.3 nM, respectively). Collectively, these results demonstrated that IgG C^1^ is a high‐quality anti‐CD30 Ab with an epitope that competes with that of CD30L and the clinical benchmark IgG BRNT.

### 2.4 Development of anti‐CD30/PD‐L1 tetravalent Abs

**FIGURE 5:**
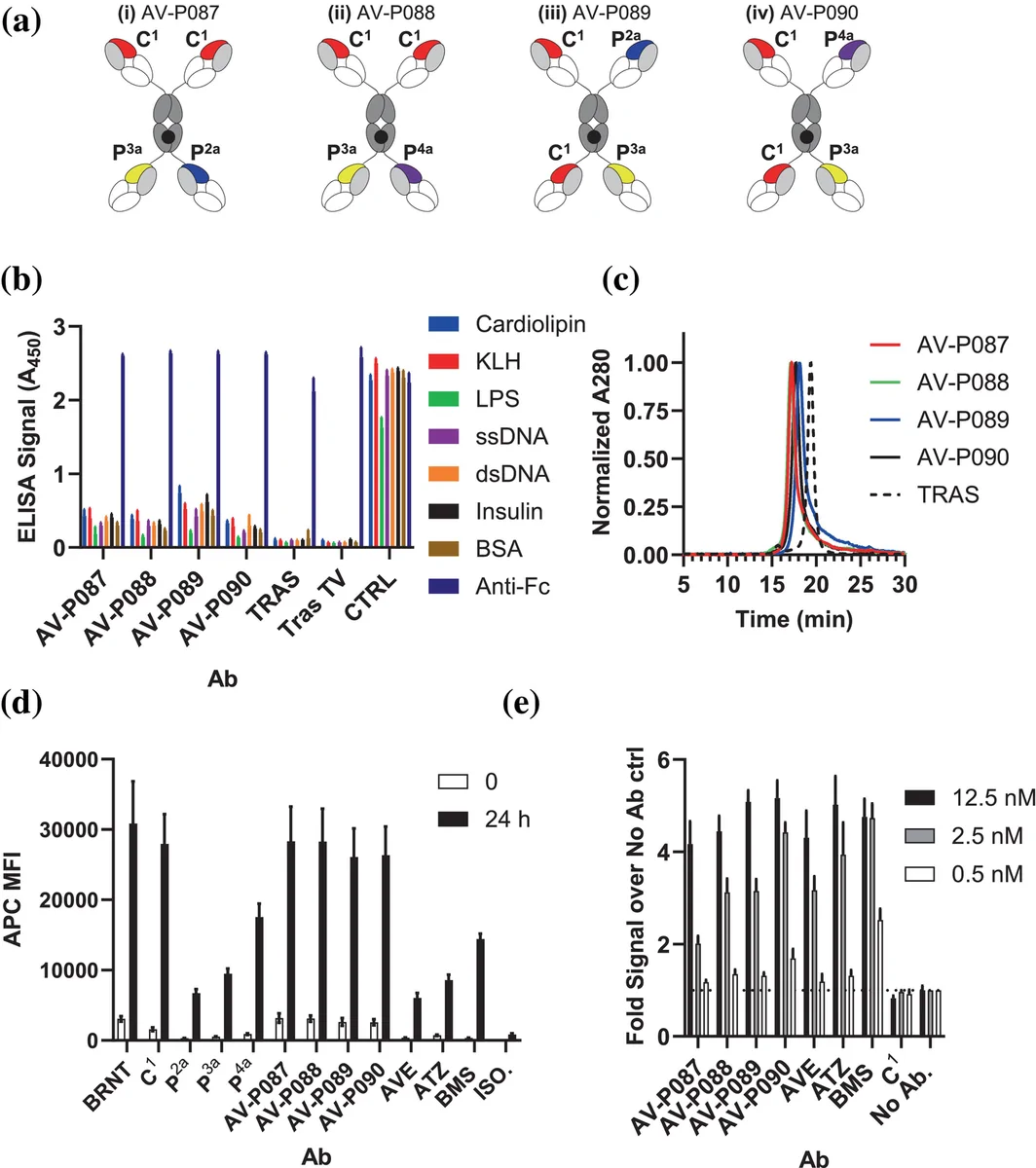
Characterization of tetravalent antibodies (Abs) targeting CD30 and PD‐L1. (a) Schematic for tetravalent bispecific Abs (i) AV‐P087, (ii) AV‐P088, (iii) AV‐P089, and (iv) AV‐P090. The domains are colored as follows: C_H1_ and C_L_, white; common V_L_, light gray; central fragment crystallizable (Fc), dark gray; mutations enabling Fc* knob‐into‐hole pairing, black circle; unique VH domains, red, blue, purple, or yellow. (b) Polyreactivity enzyme‐linked immunosorbent assay (ELISA) to detect non‐specific binding to a panel of heterologous biomolecules (*y*‐axis) for tetravalent Abs, Trastuzumab (TRAS) immunoglobulin G (IgG), and a positive control Ab (CTRL) that exhibits non‐specific binding. Abs were tested at 100 nM. (c) Analytical gel filtration size exclusion chromatography of tetravalent Abs and TRAS IgG. (d) Internalization (*y*‐axis) of 25 nM Abs (*x*‐axis) by Karpas‐299 cells, measured directly after Ab addition (0 h) or 24 h after Ab addition (24 h). Data are presented as the average from four independent experiments. (e) PD‐L1 signaling cell‐based reporter assay. Jurkat T cells stably expressing human PD‐1 and an NFAT‐driven luciferase reporter were co‐cultured with a PD‐L1‐expressing CHO‐K1 cell line. The cultures were treated with Abs (*x*‐axis) at the indicated concentrations and luciferase activity was measured by luminescence and normalized to signal without Ab treatment (*y*‐axis). Inhibition of PD‐L1 signaling results in an increase in luciferase activity. Data are presented as the average from three independent experiments. ATZ, Atezolizumab; AVE, Avelumab; BMS, BMS‐936559; BRNT, Brentuximab.

To demonstrate the utility of our modular strategy for engineering tetravalent Abs, we developed Abs that could simultaneously target PD‐L1 and CD30, which are both implicated in cHL. CD30 is a member of the tumor necrosis factor receptor (TNFR) superfamily that is expressed on >98% of cHL tumors, and an Ab‐drug conjugate targeting CD30 (BRNT‐Vedotin) is highly efficacious for treating cHL (Pro et al., 2012; Younes et al., 2010). Notably, cHL tumors are comprised of a relatively small number of malignant Reed‐Sternberg (RS) cells surrounded by a much larger population of non‐tumor inflammatory immune cells. To survive in this complex tumor microenvironment, RS cells have evolved several mechanisms to evade immune surveillance, including amplification of PD‐L1 expression (Ansell, 2017). Recently, anti‐PD‐1 Abs have been shown to be highly efficacious for treating cHL (Benevolo Savelli et al., 2024; Jaworek & Brockelmann, 2025). Thus, we designed four tetravalent Abs with two arms targeting CD30 and two arms targeting PD‐L1 as a bispecific agent that could target both receptors directly on cHL cells (Figure 5a).

In the tetravalent Fab‐Fc*‐Fab format, the central heterodimeric Fc* domain presents two Fabs fused to its N‐termini (as in natural IgG Abs) and two Fabs fused to its C‐termini (non‐natural aspect of the design; Figure 1c). We designed four tetravalent Fab‐Fc*‐Fab molecules (AV‐P087, AV‐P088, AV‐P089, and AV‐P090), which each contained two anti‐CD30 C^1^ Fabs and two anti‐PD‐L1 Fabs arranged in various geometries. AV‐P087 and AV‐P089 have two Fabs that could bind simultaneously to PD‐L1 (Figure 3f), as we speculated that this may further enhance binding to cell‐surface antigens. These Abs were produced in high yield from expression in CHO cells (270, 115, 65, and 115 mg of protein per liter of cell culture for AV‐P087, AV‐P088, AV‐P089, and AV‐P090, respectively) and were purified to near homogeneity by Protein‐A affinity chromatography, as evidenced by SDS‐PAGE (Figure S1c). The tetravalent Abs exhibited reasonable binding signals in the polyreactivity ELISA (Figure 5b), although they were slightly higher for the tetravalent Abs than for the benchmark TRAS, either in IgG or in a similar tetravalent format (TRAS TV). By analytical SEC, the purified tetravalent Abs showed a predominant monodisperse peak that eluted slightly earlier than TRAS IgG, as expected based on the larger molecular weight (Figure 5c).

Analysis of binding kinetics by BLI showed that all four tetravalent Abs bound to the human CD30 extracellular domain (ECD) with high affinities comparable to that of IgG C^1^ (Table 1). Although still binding tightly, affinities for recombinant human PD‐L1 ECD were reduced as compared with the respective IgGs.

As an initial assessment of the suitability of these tetravalent Abs to act as delivery vehicles for cytotoxic drugs, we performed internalization assays with the human T cell lymphoma line Karpas‐299 (Figure 5d). This assay is based on incubating test Abs with an anti‐Fc Fab fragment labeled with a pH‐sensitive dye (Nath et al., 2016). The dye exhibits low fluorescence at physiological pH, such as is commonly found in tissue culture media, but is highly fluorescent in the acidic environment of the endosome, thus making it an efficient readout of Ab internalization in living cells. As expected (Doronina et al., 2003), we observed strong internalization of the anti‐CD30 IgGs BRNT and C^1^ in this assay. Consistent with a previous study of ATZ (Xiao et al., 2021), we also observed significant though less robust internalization of the three clinical‐stage anti‐PD‐L1 Abs (AVE, ATZ, and BMS) and our anti‐PD‐L1 IgGs P^2a^, P^3a^, and P^4a^. Most importantly, all four tetravalent Abs exhibited strong internalization that was comparable to that of BRNT and IgG C^1^ (Figure 5d).

To confirm the activity of the anti‐PD‐L1 arms of the tetravalent Abs, we used the PD‐L1 bioassay described above (Figure 5e). As expected, the anti‐CD30 IgG C^1^ had no activity, whereas the clinical anti‐PD‐L1 IgGs and our anti‐PD‐L1 IgGs potently inhibited PD‐L1 signaling. Despite the apparent decrease in affinity for the PD‐L1 ECD observed in the BLI assay (Table 1), all four tetravalent Abs exhibited strong functional activities comparable to those of the anti‐PD‐L1 IgGs. We speculate that binding to immobilized PD‐L1 ECD in the artificial BLI context may induce steric constraints that are not present with endogenous PD‐L1 on the cell surface. Alternatively, the avidity afforded by co‐targeting CD30 and PD‐L1 on the same cell might mitigate the lower affinity observed toward recombinant purified PD‐L1. Regardless, these assays showed that the tetravalent Abs are potent inhibitors of T‐cell checkpoint inhibition, and moreover, they are robustly internalized and thus are likely to be good carriers for drug conjugates. Conjugation of these Abs with a toxic payload would likely provide a potent dual activity biologic that can simultaneously deliver cytotoxic drugs and inhibit the PD‐L1/PD‐1 immune checkpoint pathway to relieve suppression of T‐cell activity. While generating and testing antibody‐drug conjugates based on these molecules is beyond the scope of this article, we are currently evaluating the therapeutic potential of these dual‐purpose biologics.

### 2.5 The crystal structures of Fabs P^2a^, P^3a^, and P^4a^ in complex with PD‐L1

**FIGURE 6:**
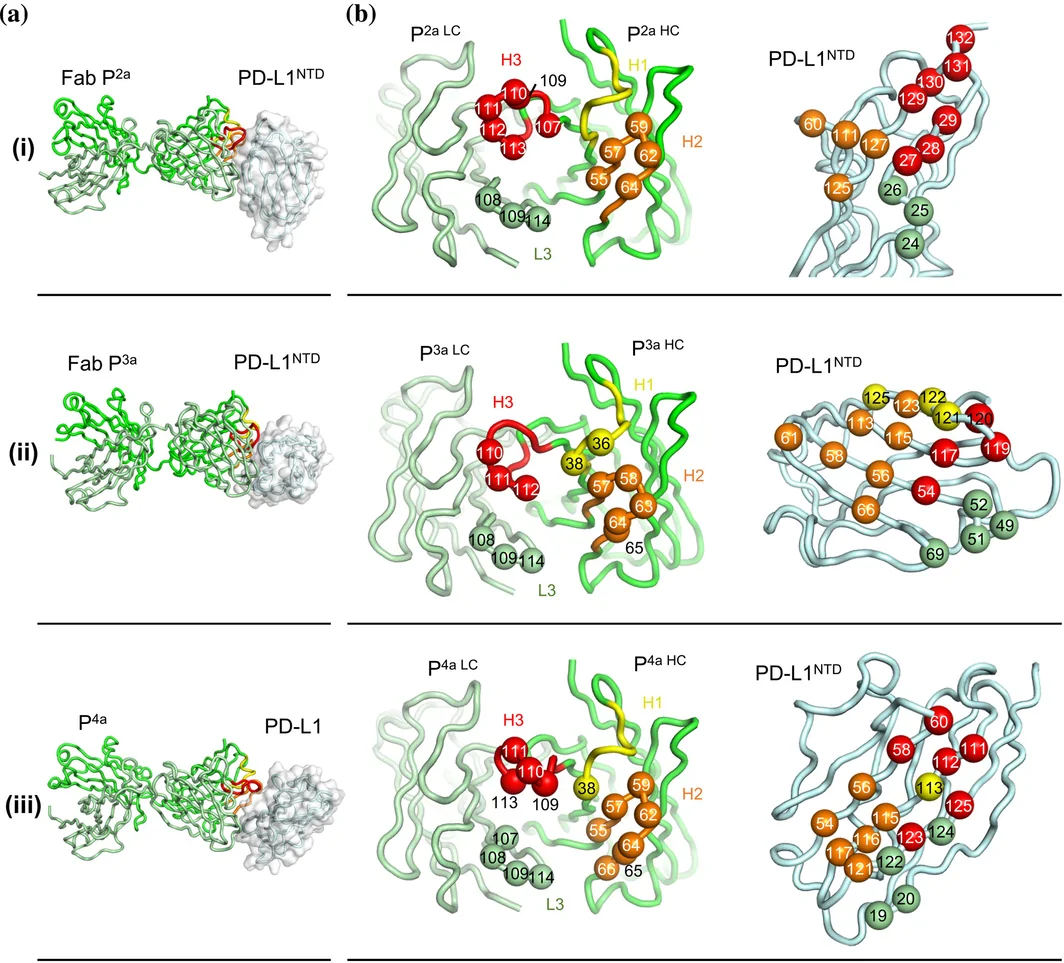
The structures of antigen‐binding fragments (Fabs) bound to PD‐L1. (a) The crystal structures of (i) Fab P^2a^, (ii) Fab P^3a^, and (iii) Fab P^4a^ bound to the N‐terminal IgV domain of the PD‐L1 extracellular domain (PD‐L1^NTD^, PDB codes 13FL, 13DS, 13DT, respectively). The main chains of the Fab light and heavy chains are colored light or dark green, respectively, and complementarity determining regions (CDRs) H1, H2, and H3 are colored yellow, orange, or red, respectively. The PD‐L1^NTD^ is shown with the surface and main chain colored gray or cyan, respectively. (b) Open book view of the interfaces between Fab (left) and PD‐L1^NTD^ (right) for (i) Fab P^2a^, (ii) Fab P^3a^, and (iii) Fab P^4a^. Contact residues within <4 Å are shown as Cα spheres numbered according to the International Immunogenetics Information System (IMGT) nomenclature for the Fab and the PDB deposition for PD‐L1^NTD^. Fab residues are colored as in (a) and PD‐L1^NTD^ residues are colored according to the region of the Fab with which they make the most contacts.

To understand the basis for the antagonist activities of the anti‐PD‐L1 Fabs, we used x‐ray crystallography to determine the structure of each Fab in complex with the N‐terminal IgV domain of PD‐L1 (PD‐L1^NTD^). The Fabs were designed using an engineered Fab framework, S1CE2, that we previously optimized to enhance crystallization (Bruce, Singer, Blazer, et al., 2024; Bruce, Singer, Filippova, et al., 2024). The complexes with Fabs P^2a^, P^3a^, and P^4a^ crystallized in space groups C222_1_, P2_1_2_1_2_1_, and P2_1_ at resolutions of 2.0, 1.9, and 1.6 Å and with *R*_free_ values of 24%, 21%, and 20%, respectively (Protein Databank IDs 13FL; 13DS; and 13DT, respectively). The asymmetric unit for each complex contained one Fab and one protomer of PD‐L1^NTD^ (Figure 6a,i–iii). The epitopes for the three Fabs were all on one face of PD‐L1^NTD^ and complexation resulted in the burial of 743, 821, and 811 Å^2^ of surface area on Fabs P^2a^, P^3a^, and P^4a^, respectively. For each Fab, the heavy chain contributed the majority of the buried surface area (74%, 76%, and 79% for Fabs P^2a^, P^3a^, and P^4a^, respectively; Table S1). Most contacts within each paratope were with CDRs H2 and H3 in the heavy chain, supported by contacts with CDR‐L3 in the light chain (Figure 6b,i–iii). Below, we describe the molecular details of the interactions of the CDRs of each Fab with PD‐L1^NTD^.

**FIGURE 7:**
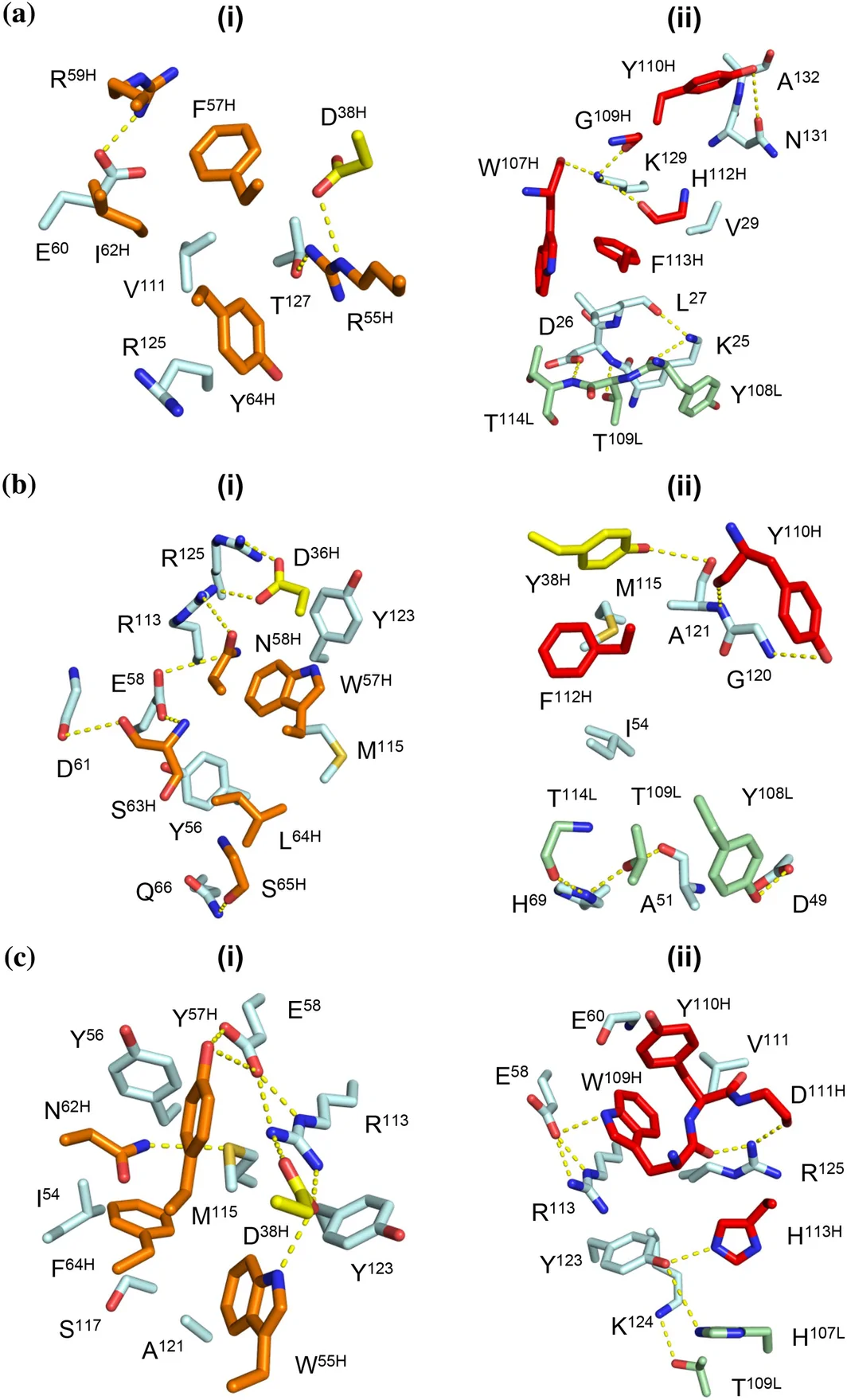
Molecular details of the contacts between Fabs and PD‐L1. Side chain interactions with PD‐L1 (cyan) are shown for (a) Fab P^2a^, (b) Fab P^3a^, and (c) Fab P^4a^ for (i) CDR‐H2 (orange) and CDR‐H1 (yellow) and for (ii) CDR‐H1 (yellow), CDR‐H3 (red), and CDR‐L3 (green). Salt‐bridges and hydrogen bonds are shown as dashed yellow lines. CDR, complementarity determining regions.

In Fab P^2a^, CDR‐H2 residues Phe^57H^, Ile^62H^, and Tyr^64H^ form a hydrophobic patch that interacts with PD‐L1^NTD^ residue Val^111^ (Figure 7a,i). This is supported by a hydrogen bond between Thr^127^ and Arg^55H^, which in turn is coordinated by hydrogen bonding with Asp^38H^ in CDR‐H1. This interaction, together with a salt‐bridge between Arg^59H^ and Glu^60^, supports the hydrophobic interactions with Val^111^. CDR‐H3 mediates extensive contacts including hydrogen bonds that the backbone carbonyls of Trp^107H^, Gly^109H^, and His^112H^ form with the side chain of Lys^129^ (Figure 7a,ii). Coordination of these residues allows for extensive hydrophobic interactions through packing of Phe^113H^ into a hydrophobic pocket formed by Leu,^27^ Val,^29^ and Lys^129^. Leu^27^ in turn packs into a hydrophobic pocket between Trp^107H^ and Phe^113H^. In addition, Tyr^110H^ hydrogen bonds with Asn^131^ and packs along Ala^132^. In CDR‐L3, the backbone carbonyl of Tyr^108L^ and amine of Thr^114L^ form hydrogen bonds with the side chains of Lys^25^ and Asp,^26^ respectively, and the side chain of Thr^109L^ forms a hydrogen bond with the backbone amine of Asp.^26^ In addition, the side chains of Tyr^108L^ and Thr^114L^ pack against the side chains of Lys^25^ and Leu,^27^ respectively.

In Fab P^3a^, CDR‐H2 is shorter than intended in the library design, but nonetheless, it provides most of the contacts contributed by the heavy chain. Leu^64H^ and Trp^57H^ from CDR‐H2 pack against a hydrophobic surface formed by Tyr^56^, Met^115^, and Tyr^123^ (Figure 7b,i). CDR‐H2 also forms hydrogen bonds between the side chain of Asn^58H^ and Arg^113^ and Glu^58^, the side chain of Ser^63H^ and Asp^61^, the backbone amine of Ser^63H^ and Glu^58^, and the backbone carbonyl of Ser^65H^ and Gln^66^. In CDR‐H1, Asp^36H^ forms salt‐bridges with Arg^113^ and Arg^125^ (Figure 7b,i), and the side chain of Tyr^38H^ forms a hydrogen bond with the backbone carbonyl of Ala^121^ (Figure 7b,ii). Tyr^38H^ also packs along a hydrophobic surface formed by Met^115^ and Ala^121^. In CDR‐H3, the side chain hydroxyl and backbone carbonyl of Tyr^110H^ form hydrogen bonds with the backbone amines of Gly^120^ and Ala^121^, respectively, and Phe^112H^ packs into a hydrophobic pocket formed by Met^115^, Ala^121^, and Ile^54^. Finally, in CDR‐L3, the side chain of Tyr^108L^ hydrogen bonds with the side chain of Asp^49H^, Thr^109L^ hydrogen bonds with the side chain of His^69^ and the main chain carbonyl of Ala^51^, and the side chain of Thr^114L^ hydrogen bonds with His^69^.

In CDR‐H2 of Fab P^4a^, Asn^62H^ hydrogen bonds with Met^115^ and packs along Tyr^56^, and the side chain of Tyr^57H^ forms a hydrogen bond with Glu^58^, which orients the aromatic ring of Tyr^57H^ to make a cation‐pi interaction with Arg^113^. In addition, Tyr^57H^, Phe^64H^, and Trp^55H^ pack along a hydrophobic surface formed by Ile^54^, Met^115^, Ser^117^, Ala^121^, and Tyr^123^ (Figure 7c,i). In CDR‐H1, the side chain of Asp^38H^ forms a salt‐bridge with the side chain of Arg^113^ (Figure 7c,i). The CDR‐H3 backbone carbonyls of Trp^109H^ and Asp^111H^ hydrogen bond with Arg^125^ (Figure 7c,ii). This opens a hydrophobic pocket between the main chain of Glu^60^ and the side chains of Val^111^, Arg^113^, and Arg^125^, into which side chains of Trp^109H^ and Tyr^110H^ pack. These interactions are supported by hydrogen bonding between Trp^109H^ and Glu^58^, between His^113H^ and Tyr^123^, and in CDR‐L3, between His^107L^ and Tyr^123^ and between Thr^109L^ and Lys^124^.

### 2.6 Comparison of PD‐L1 interactions with Fabs P^2a^, P^3a^, and P^4a^, and with PD‐1

**FIGURE 8:**
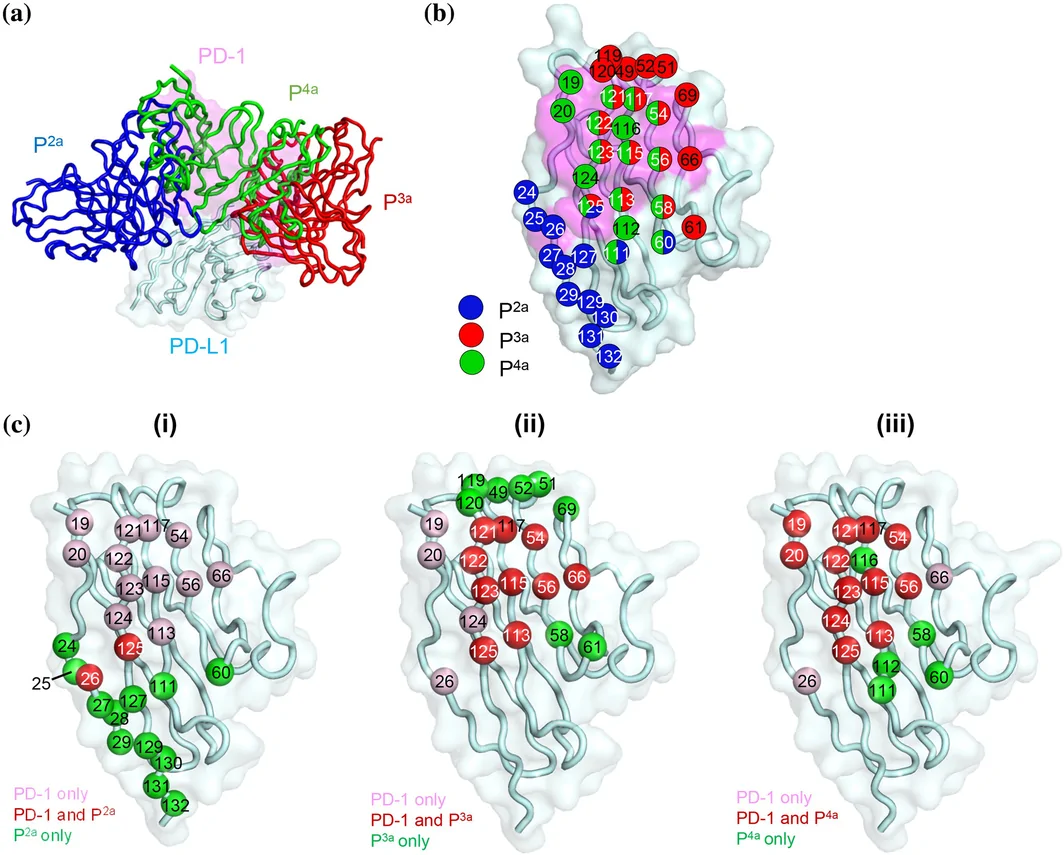
Comparison of PD‐L1 interactions with PD‐1 and with antigen‐binding fragments (Fabs) P^2a^, P^3a^, and P^4a^. (a) Superposition of the structures of the PD‐L1^NTD^ (cyan surface) in complex with PD‐1 (magenta surface, PDB entry 3BIK), Fab P^2a^ (blue), Fab P^3a^ (red), and Fab P^4a^ (green). For clarity, only the PD‐L1^NTD^ bound to PD‐1 is shown as a cartoon with a transparent surface and the main chains of Fabs (only Fv domains) are shown as cartoons. (b) Schematic representation of the PD‐L1^NTD^ with residues that contact Fabs shown as circles colored as follows: P^2a^ (blue), P^3a^ (red), and P^4a^ (green). The PD‐L1^NTD^ is shown as a cyan surface with residues buried in the PD‐1 complex colored magenta. (c) Comparison of the PD‐1 binding site on PD‐L1^NTD^ with the epitopes for (i) Fab P^2a^, (ii) Fab P^3a^, and (iii) Fab P^4a^. The PD‐L1^NTD^ main chain and surface are colored cyan. Residues that contact PD‐1 and/or Fab are shown as spheres colored as follows: PD‐1 only (pink), Fab only (green), PD‐1 and Fab (red). Numbering is according to UniProt Q9NZQ7‐1.

Superposition of the structures of the three Fabs bound to PD‐L1^NTD^ (Figure 8a) was consistent with the epitope binning data determined by competitive BLI (Figure 3f). Fabs P^2a^ and P^3a^ bind to distinct regions of PD‐L1^NTD^ and do not sterically interfere with each other. In contrast, Fab P^4a^ blocks both Fabs P^2a^ and P^3a^ by binding to a central epitope. Analysis of the interface residues on PD‐L1 recognized by each paratope (Figure 8b) reveals that the majority of contacts for P^2a^ are distinct, with only one or three residues overlapping with the P^3a^ or P^4a^ epitopes, respectively. In contrast, the P^3a^ and P^4a^ epitopes overlap extensively and share 10 residues.

To understand the basis for inhibition of the PD‐L1/PD‐1 interaction, we also compared the epitope of each Fab with the PD‐1 binding site on PD‐L1 (Figure 8c). The Fab P^2a^ epitope overlaps only slightly with the PD‐1 binding site, sharing only two contact residues (Figure 8c,i). In contrast, the Fab P^3a^ epitope (Figure 8c,ii) and the P^4a^ epitope (Figure 8c,iii) overlap extensively with the PD‐1 binding site, sharing 10 or 12 contact residues, respectively. Thus, despite recognizing different epitopes with different degrees of overlap with each other and the PD‐1 binding site, direct steric hindrance can explain the ability of each Ab to activate T‐cell signaling by inhibiting the PD‐L1/PD‐1 interaction.

### 2.7 The crystal structure of fab C^1^ in complex with CD30

**FIGURE 9:**
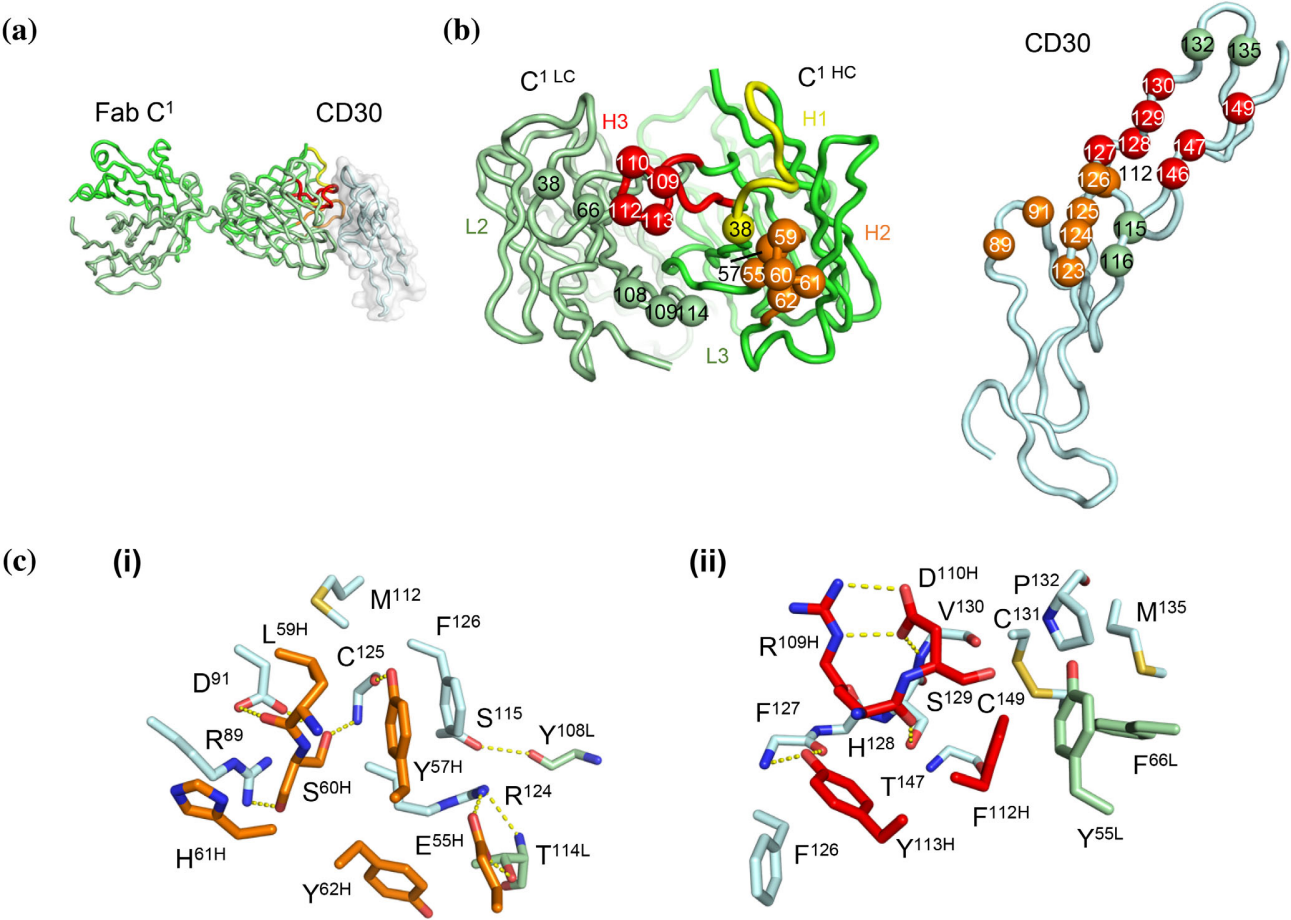
The structure of antigen‐binding fragment (Fab) C^1^ bound to CD30. (a) The crystal structure of Fab C^1^ bound to CD30 (PDB code 13HA). The Fab light and heavy chains are colored light or dark green, respectively, and the complementarity determining region (CDRs) H1, H2, and H3 are colored yellow, orange, or red, respectively. CD30 is shown as a gray surface with its main chain colored cyan. (b) Open book view of the interfaces between Fab C^1^ (left) and CD30 (right). Contact residues within 4 Å are shown as Cα spheres numbered according to the International Immunogenetics Information System (IMGT) nomenclature for the Fab and the PDB deposition for CD30, respectively. Fab residues are colored as in (a) and CD30 residues are colored according to the region of the Fab with which they make the most contacts. (c) Molecular details of the contacts between Fab C^1^ and CD30 (cyan) for (i) CDR‐H2 (orange), CDR‐H1 (yellow), and CDR‐L3 (green) and for (ii) CDR‐H3 (red), and CDR‐L2 (green). Salt‐bridges and hydrogen bonds are shown as dashed yellow lines.

We solved the structure of Fab C^1^ in complex with the second and third cysteine‐rich domains (CRDs) (residues 66–153, UniProt P28908‐1) of CD30 (CD30^CRD2/3^) by x‐ray crystallography, again using the Fab framework (S1CE2) optimized to enhance crystallization (Bruce, Singer, Blazer, et al., 2024; Bruce, Singer, Filippova, et al., 2024). The complex crystallized in space group P3_1_21 at a resolution of 2.0 Å and an *R*_free_ value of 22.2% (PDB ID 13HA). The asymmetric unit for the complex contained one complete Fab heterodimer and one protomer of CD30^CRD2/3^ (Figure 9a). Complexation resulted in the burial of 807 Å^2^ of surface area on Fab C^1^. On the Fab, most of the buried surface area was contributed by the heavy chain (77%; Table S1). Most of the epitope/paratope contacts involve CDRs H2 and H3, with additional supporting contacts involving CDRs H1, L2, and L3 (Figure 9b).

CDR‐H2 forms a series of hydrogen bonds with CD30^CRD2/3^ through the backbone carbonyls of Leu^59H^ and Ser^60H^ with the side chains of Asp^91^ and Arg^89^, respectively (Figure 9c,i). In addition, Tyr^57H^ hydrogen bonds with the backbone carbonyl of Cys^125^, and the side chain of Ser^60H^ hydrogen bonds with the backbone amine of Cys^125^ and the side chain of Asp^91^. Glu^55H^ forms a salt‐bridge with the side chain of Arg^124^ and hydrogen bonds with Thr^114L^, which in turn hydrogen bonds with Arg^124^ through the backbone amine. In combination with these hydrogen bonds, the interface involves hydrophobic interactions between His^61H^ and Arg^89^, Tyr^62H^ and Arg^124^; Tyr^57H^ and Phe^126^; and Leu^59H^ with Asp^91^, Met^112^, and Cys^125^. Along with the hydrogen bonds with Thr^114L^ from CDR‐L3, Tyr^108L^ hydrogen bonds with the side chain of Ser^115^. In CDR‐H3 (Figure 9c,ii), the side chain of Asp^110H^ hydrogen bonds with the backbone amine of Val^130^. In addition, the side chain of Tyr^113H^ hydrogen bonds with the backbone amine and carbonyl of Phe^127^, positioning the side chain to also form hydrophobic interactions with Phe^126^. The backbone carbonyl of Arg^109H^ hydrogen bonds with the side chain of Ser^129^, and the side chain of Arg^109H^ forms a salt‐bridge with the side chain of Asp^110H^ and packs along the backbone of Ser^129^ and His^128^. Together with CDR‐L2 residues Tyr^55L^ and Phe^66L^, Phe^112H^ packs with a hydrophobic surface formed by Ser^129^, Val^130^, Cys^131^, Pro^132^, Met^135^, and Thr^147^.

**FIGURE 10:**
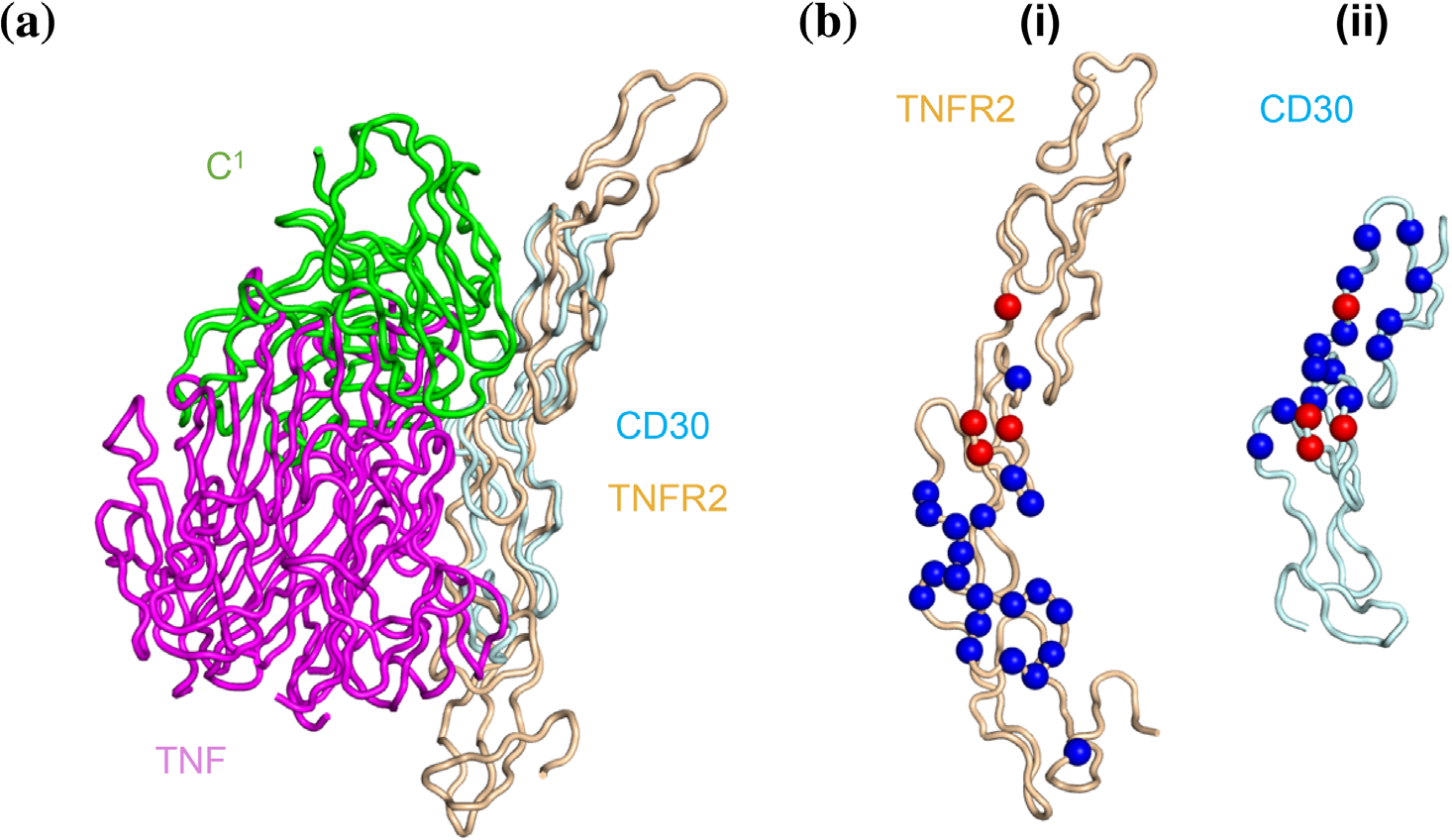
Comparison of the structures of antigen‐binding fragments (Fab) C^1^ in complex with CD30 and the TNFR2 in complex with TNF. (a) Superposition of the Fab‐C^1^:CD30^CRD2/3^ complex with the TNFR2:TNF complex (PDB ID 3ALQ). Only the Fv (green) of C^1^ is shown in complex with CD30^CRD2/3^ (cyan). TNFR2 and the TNF trimer are colored tan and magenta, respectively. (b) Comparison of the binding site of (i) TNF on TNFR2 with (ii) the epitope for Fab C^1^ on CD30. Residues on tumor necrosis factor receptor (TNFR) or CD30 that contact (<4 Å) TNF or Fab C^1^, respectively, are shown as blue Cα spheres. Contact residues that overlap between the two structures are shown as red Cα spheres.

There is no reported structure of CD30 bound to CD30L, and in fact, our structure represents the first reported structure of the CD30 ECD in any form. Since no structure exists for the CD30/CD30L complex, it is not possible to determine directly how Fab C^1^ competes with CD30L for binding to CD30. However, the interactions of members of the TNFR superfamily with their ligands are highly conserved, and thus, we took advantage of the structure of the TNFR2/tumor necrosis factor (TNF) complex (PDB ID 3ALQ) (Mukai et al., 2010) to model the CD30/CD30L complex. Superposing CD30^CRD2/3^ bound to C^1^ in our structure with the TNFR2 ECD bound to TNF revealed high similarity (PyMol align, root mean square deviation (RMSD) = 2.29 Å; Figure 10a). We then compared the CD30 residues that contact Fab C^1^ with those of TNFR2 that contact TNF (Figure 10b). This analysis revealed four overlapping residues between the CD30 residues that contact Fab C^1^ and those predicted to contact CD30L by analogy with the TNFR2/TNF interaction. AlphaFold models of CD30 and CD30L are also consistent with this analysis (Bittrich et al., 2024; Fleming et al., 2025; Jumper et al., 2021). In particular, our CD30 structure aligns well with the AlphaFold models of both CD30 and TNFR2 (CD30: AF‐P28908‐F1‐v6, residues 69–152, RMSD 1.37 Å, and TNFR2: AF‐P20333‐F1‐v6, residues 78–163, RMSD 1.86 Å) and the TNF structure aligns well with the CD30L model (CD30L: AF‐P32971‐F1‐v6, residues 105–215, RMSD 1.32 Å; Figure S2). Thus, our structural data are consistent with Fab C^1^ inhibiting the binding of CD30L via direct steric hindrance.

### 2.8 Structural analysis of the common light chain in diverse molecular interactions

**FIGURE 11:**
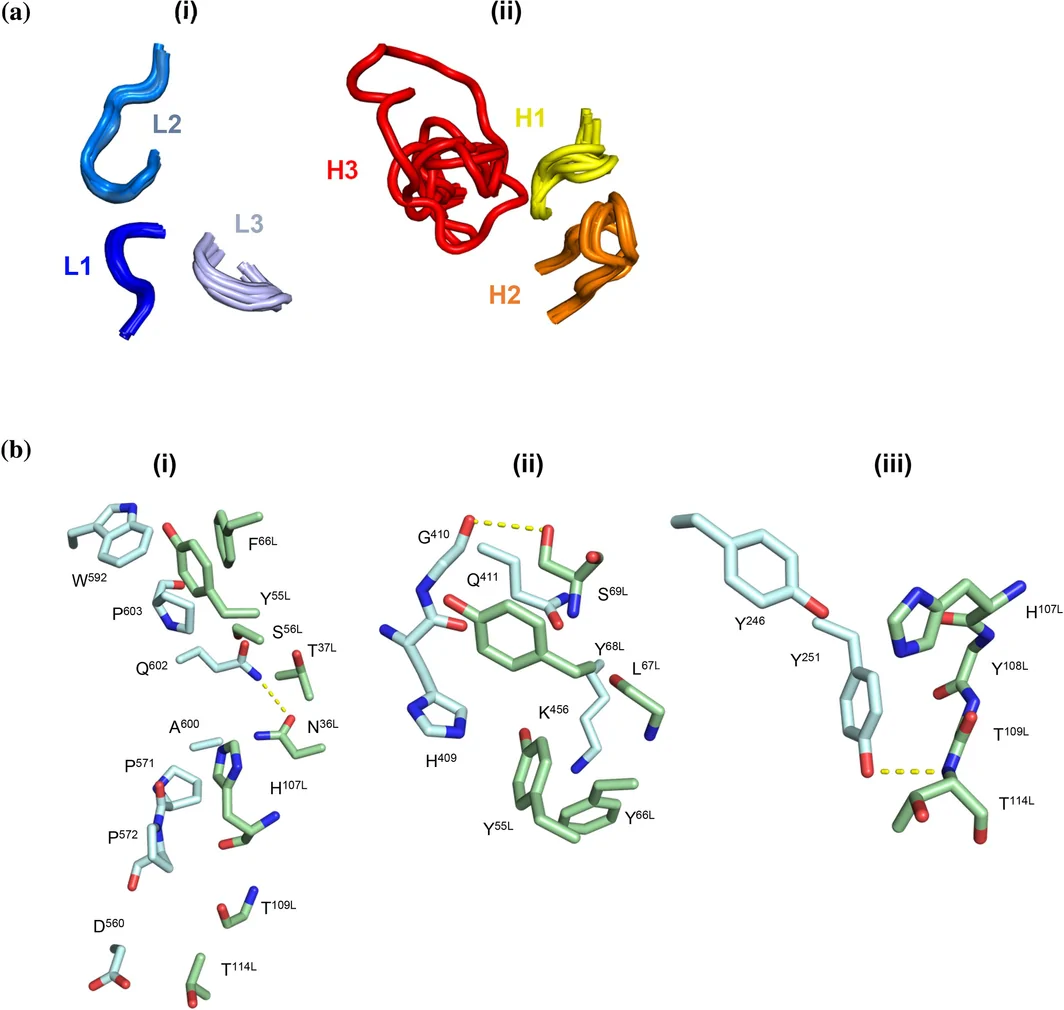
Comparison of complementarity determining region (CDR) conformations for antigen‐binding fragments with the common Trastuzumab (TRAS) light chain. (a) Superposition of (i) light chain CDRs L1 (blue), L2 (azure blue), and L3 (lavender) and (ii) heavy‐chain CDRs H1 (yellow), H2 (orange), and H3 (red) from the structural alignment of Fabs TRAS (PDB code 8PWH), ER‐2a (PDB code 9Z9F), ER‐3c (PDB code 9Z9E), P^2a^, P^3a^, P^4a^, and C^1^. (b) Molecular details of interactions between the light chain (green) and antigen (cyan) for (i) TRAS CDRs L1, L2, and L3 with HER2, (ii) ER‐3c CDR‐L2 with EGFR, and (iii) ER‐2a CDR‐L3 with EGFR. Hydrogen bonds are shown as dashed yellow lines.

In addition to shedding light on individual molecular interactions, our structural analysis provided the rare opportunity to observe how a common light chain functions in seven distinct paratopes, namely: the anti‐HER2 Fab TRAS, two previously reported anti‐epidermal growth factor receptor (EGFR) Fabs (ER‐2a and ER‐3c) (Adams et al., 2026), the anti‐PD‐L1 Fabs P^2a^, P^3a^, and P^4a^ (Figure 6), and the anti‐CD30 Fab C^1^ (Figure 9). Structural alignment (PyMol align) of the light chains revealed high similarity, aligning to the parental TRAS light chain with an RMSD of <0.87 Å and having less than 2.1 Å deviation between Cα atoms of all light‐chain CDRs (Figure 11a,i). Alignment of the heavy chains (PyMol align, RMSD <2.1 Å) showed high conformational diversity in CDR‐H3, while CDRs H1 and H2 remained similar with <3.2 and <4.9 Å deviation between Cα atoms, respectively (except the shorter CDR‐H2 of Fab P^3a^, Figure 11a,ii).

In the parental TRAS paratope (PDB code 8PWH), the light chain makes a large portion of the contacts with HER2, contributing 38% of the total 863 Å^2^ buried surface area. In the Fabs with the common TRAS light chain that bind to other antigens, a total of 743–1195 Å^2^ of surface area was buried in the diverse epitopes on EGFR, PD‐L1, and CD30. As would be expected from the library design, the heavy‐chain CDRs made extensive contacts with each of the epitopes. Although the residues remained constant, in all cases the light chains of Fabs ER‐2a, ER‐3c, C^1^, P^2a^, P^3a^, and P^4a^ contributed significantly to the total buried surface area (10%, 19%, 23%, 26%, 24%, and 22%, respectively). The light chain of TRAS contributes contacts from all three light‐chain CDRs in its engagement with HER2 (Figure 11b,i), through a series of van der Waals interactions with CDR‐L2 (Ser^56L^, Tyr^55L^, and Phe^66L^ with Trp^592^ and Pro^603^) and CDR‐L3 (Thr^114L^, Thr^109L^, and His^107L^ with Asp^560^, Pro^572^, and P^571^, respectively), and through a hydrogen bond between Gln^602^ and Asn^36L^ of CDR‐L1. The same light chain in the context of the anti‐PD‐L1, ‐EGFR, and ‐CD30 Fabs also makes contacts with their respective antigens, albeit fewer than observed with the parental TRAS. In the extensive interface between Fab ER‐3c and EGFR, CDR‐L2 contributed all contacts made by the light chain (Figure 11b,ii). Similarly, Tyr^55L^ and Phe^66L^ in CDR‐L2 of Fab C^1^ make van der Waals contacts with CD30 (Figure 9c,ii). Of particular interest are the molecular interactions mediated by CDR‐L3 residues His^107L^, Tyr^108L^, Thr^109L^, and Thr^114L^. These four residues are highly adaptable to the epitope topography, making extensive contacts with diverse epitopes through van der Waals interactions and hydrogen bonds in most of the structures analyzed (Figure 7a,ii [P^2a^], Figure 7b,ii [P^3a^], Figure 7c,ii [P^4a^], Figure 9c,i [C^1^], and Figure 11b,iii [ER‐2a]). As discussed below, in Fab ER‐3c, these four residues do not make direct contact with EGFR but do function to support the conformation of CDR‐H3. Between the adaptable contacts mediated by the light‐chain CDRs and the extensive contacts mediated by the heavy‐chain CDRs, these results show that the fixed light chain does not appear to limit the range of epitopes that can be recognized by Fabs derived from our naive heavy‐chain library.

In addition to contacts with the epitope, CDRs L2, L3, and to a limited extent L1, provide structural support for positioning CDR‐H3 to make favorable contacts with each antigen (Figure S3). The rigid light‐chain CDRs, with alternating surface exposed weakly‐polar and non‐polar residues, provide a neutral landscape for diverse interactions with the variable CDR‐H3 residues. Contrary to standard selection strategies where both heavy‐ and light‐chain CDRs are varied cooperatively, by only varying the heavy‐chain CDRs, the selection process simultaneously optimizes CDR‐H3 for contacts with the antigen and the fixed light chain. This is best exemplified by Fabs ER‐2a and ER‐3c (Figure S3a,b, respectively), in which CDR‐H3 is extended and forms the majority of the heavy‐chain contacts with the epitope and extensive contacts with all three light‐chain CDRs. The shorter CDR‐H3 loops of Fabs C^1^, P^2a^, P^3a^, and P^4a^ (Figure S3c–f, respectively) all contact Tyr^55L^ and His^107L^ at minimum, with additional contacts with one or more residues from either CDR‐H2 or CDR‐H3. Thus, the light‐chain CDRs support the conformational diversity of the CDR‐H3 loops, providing the plasticity required to conform to diverse epitopes in the context of a fixed light chain.

## 3 CONCLUSIONS

In recent decades, Abs have become a class of therapeutics that substantially benefit human health. While a monospecific IgG is the standard modality in clinical applications, there is significant value in expanding the pharmacological breadth of these molecules. In particular, bispecific and multispecific Abs can exhibit activities that extend beyond the range of conventional monospecific Abs. However, development of Abs with multiple specificities adds complexity during the development process, including the potential for mispairing between heavy and light chains. To circumvent these issues, we developed a synthetic library F with a fixed light chain. Fabs derived from this library can be readily combined into tetravalent, multispecific Abs. To demonstrate the utility of this strategy, we previously developed two Fabs targeting distinct epitopes on EGFR and showed that dual paratopes enhanced the efficacy of a tetravalent Ab beyond the scope of monospecific Abs in cancer cell lines that depend on EGFR for proliferation (Adams et al., 2026).

Here, we further demonstrated the broad utility of the strategy by developing three unique Fabs targeting PD‐L1 and one Fab targeting CD30. For each Ab, we demonstrated functional activity using biochemical and cellular assays. Most importantly, we showed that these paratopes can be recombined in a modular fashion to assemble tetravalent, multispecific Abs that retain the individual functions of the parental Fabs but exhibit enhanced activities as a result of cooperativity between the Fabs in the tetravalent context. Structural analyses revealed how a common light chain can cooperate with different heavy chains to mediate interactions with diverse antigens. The insights gained from these analyses are being used to guide the development of even more functional Ab libraries with fixed light chains.

## 4 METHODS

### 4.1 Cell lines

Mammalian cells were maintained in a humidified environment at 37°C under 5% CO_2_ in the indicated media: Expi293F (ThermoFisher A14527) were grown in Expi293F medium (ThermoFisher); L540 (DSMD CellDive ACC 72) were grown in Roswell Park Memorial Institute (RPMI) 1640 (Gibco A10491‐01) supplemented with 20% fetal bovine serum (FBS) (Gibco A3160702). Karpas 299 cells were maintained in RPMI supplemented with 2 mM glutamine and 10% FBS and penicillin/streptomycin.

### 4.2 Antibody selections

Fab‐Phage libraries were designed, constructed, and validated using previously described methods (Adams et al., 2014; Giang et al., 2023; Persson et al., 2013). Selections were performed using previously established methods (Adams et al., 2014; Fellouse et al., 2007; Persson et al., 2013). Briefly, 96‐well Nunc Maxisorp plates (Thermo Fisher 12565135) were coated with either human CD30 (R&D Biosystems, 6126‐CD‐100) or, for PD‐L1 selections, with Neutravidin (ThermoFisher, 31000) at 5 μg/mL in phosphate‐buffered saline (PBS) for 16 h at 4°C. Coated plates were blocked for 2 h at ambient temperature in PBS supplemented with 0.5% bovine serum albumin. Purified phage libraries (50 μL; ~10^12^ phage/mL) were precleared for 1 h at ambient temperature on wells coated with Neutravidin to remove non‐specific binders from the library. For CD30 selections, phage were then transferred to wells containing CD30 and incubated for 1 h. For PD‐L1 selections, phage were mixed with a dilution of biotinylated human PD‐L1 (R&D Biosystems BT156‐050) for 1 h and then added to Neutravidin‐coated wells for 30 min. After incubation, the wells were extensively washed with PBS, 0.05% Tween‐20. For affinity maturation, the concentration of antigen was reduced to increase the stringency of the selection. Phage were eluted with 0.1M HCl for 10 min at ambient temperature. Eluted phage were neutralized in 1M Tris pH 8.0 and amplified as previously described for the next round of selections (Adams et al., 2014).

### 4.3 Antibody production

IgG proteins were produced in transiently transfected Expi293 cells and purified using Protein‐A affinity chromatography as previously described (Adams et al., 2025; Miersch et al., 2021; Tao et al., 2019). Briefly, plasmids encoding heavy and light chains were co‐transfected into Expi293 cells using FectoPro (Sartorius; 101000007). After 5 days, the cultures were clarified via centrifugation and the supernatant was applied to an rProtein‐A affinity column (Cytiva GE17‐1279‐03). The column was washed with PBS and retained protein was eluted with IgG elution buffer (Pierce; 21004) and immediately neutralized to pH 8.0 by the addition of Tris‐NaCl. Ab fractions were combined, concentrated, and buffer exchanged into PBS, pH 7.4. Abs were characterized by SDS‐PAGE and concentration was determined by spectrophotometry at an absorbance of 280 nm. Tetravalent Abs AV‐P087, AV‐P088, AV‐P089, and AV‐P090 were produced as custom proteins by Celltheon Expression Technologies (Union City, CA) via transient expression in CHO cells.

### 4.4 Analytical SEC

Analytical SEC was performed as previously described (Adams et al., 2025). Briefly, samples were diluted to 1 mg/mL in PBS. Each sample (50 μL) was centrifuged at 14,000 rpm for 10 min and the supernatant was transferred into an high‐performance liquid chromatography glass vial (Sigma‐Aldrich 29122‐U). Each sample (20 μL) was then separated on a Tosoh TSKgel G3000SWxl analytical SEC column fitted with a guard column in a mobile phase consisting of filtered and de‐gassed PBS. The separation analysis proceeded for 30 min at a flow rate of 0.5 mL/min while monitoring absorbance at 280 nm.

### 4.5 Binding assays

Polyreactivity ELISAs were performed as previously described (Adams et al., 2025) with modifications. A 384‐well maxisorp plate was coated overnight at 4°C with the indicated antigens (cardiolipin, 50 mg/mL; keyhole limpet hemocyanin , 5 mg/mL; lipopolysaccharide, 10 mg/mL; single‐stranded deoxyribonucleic acid, 1 mg/mL; double‐stranded deoxyribonucleic acid, 1 mg/mL; insulin, 1 mg/mL; bovine serum albumin (BSA), 0.5% in 1× PBS; goat anti‐human Fc, 1 mg/mL). Abs were tested at a concentration of 100 nM. All samples were run in duplicate, and experiments were independently repeated two to three times. All washing, blocking, incubations, color development and data collection were performed as previously described (Adams et al., 2025).

Specific binding ELISAs were performed in a 384‐well maxisorp plate that was coated overnight at 4°C with 1 μg/mL of the indicated antigens in PBS (human CD30, R&D Biosystems, 813‐CD; mouse CD30, R&D Biosystems, 852‐CD; rat CD30, R&D Biosystems, 11034‐CD; cynomolgus monkey CD30, purified in‐house as a His‐tagged fragment corresponding to residues 1–379 of National Center for Biotechnology Information Reference Sequence XP_045236472.1). Remaining binding sites were blocked with 0.5% BSA in PBS for 1 h at ambient temperature after which the plate was washed six times with PBS, 0.05% Tween20 (PBST). Abs (100 nM) were diluted in PBST and added to the wells for 1 h. Unbound protein was removed by extensive washing and bound protein was detected using an anti‐Kappa Light Chain HRP Ab (Southern Biotech; 2060‐05). The assay was developed using 3,3',5,5'‐tetramethylbenzidine reagent and quantified by absorbance at 450 nm. Data are shown as the average from three independent experiments.

### 4.6 Biolayer interferometry assays

All BLI experiments were performed on an Octet HTX with a shake speed of 1000 rpm at 25°C. For PD‐L1 affinity measurements, Abs were immobilized on Octet AHC Fc‐capture biosensors (Sartorious; Cat. 18‐5063) in assay buffer (PBS, 1% BSA, 0.05% Tween‐20) and unoccupied binding sites were quenched with human Fc. After stabilization, biosensors were dipped into dilutions of His‐tagged PD‐L1 (R&D Biosystems; 9049‐B7; 100, 33, 11, 3.7, or 1.2 nM) and association was monitored for 600 s. Then, biosensors were moved to buffer‐containing wells and dissociation was monitored for an additional 600 s. Data for each experiment were fitted using a 1:1 global fitting model to determine kinetic constants. Data are shown from three independent experiments.

For CD30 affinity measurements, Abs were immobilized on Octet AR2G biosensors (Sartorious; 18‐5093). Reactive sites remaining after immobilization were quenched using BSA in assay buffer. After equilibration in assay buffer, biosensors were dipped into dilutions of His‐tagged CD30 (Sino Biological; 10777‐H08H; 200, 67, 22, 7, and 2.5 nM) and association was monitored for 600 s. Then, biosensors were moved to buffer‐containing wells and dissociation was monitored for an additional 600 s. Data for each experiment were fitted using a 1:1 global fitting model to determine kinetic constants. Data are shown as the average from three independent experiments.

Ab selectivity was determined by capturing Fc‐tagged human PD‐L1, mPD‐L1, cPD‐L1, human PD‐L2, or Fc on Octet AHC Fc‐capture biosensors pre‐equilibrated with assay buffer. Unbound sites were quenched with Fc. After signal stabilization, coated biosensors were dipped into 200 nM Ab in assay buffer for 300 s. The steady‐state signal at the end of the association phase was extracted for analysis. Averaged data are shown from three independent experiments.

Epitope binning and competition experiments were performed similarly to the selectivity measurements with the following modifications. Human PD‐L1‐coated sensors were exposed to 200 nM of blocking Ab for 300 s and then immediately exposed to 200 nM of a binding Ab or Fc‐tagged human PD‐1 or human CD80 (R&D Biosystems; 10133‐B1) for an additional 300 s. Steady state signal from each binding Ab was extracted for analysis and normalized to the signal derived from equivalent sensors saturated with a non‐binding control Ab (TRAS). Similar experiments were performed for CD30 Abs. Averaged data are shown from three independent experiments.

### 4.7 PD‐L1/PD‐1 cellular inhibition assay

Abs were tested in a PD‐1/PD‐L1 blockade assay according to the manufacturer's instructions (Promega, J1255). Briefly, artificial antigen‐presenting CHO‐K1 cells engineered to express PD‐L1 and a cell‐surface protein that activates T cells in an antigen‐independent manner were incubated with Abs for 1 h at 37°C, and then co‐cultured with effector Jurkat T cells stably expressing human PD‐1 and an NFAT‐driven luciferase reporter that is suppressed by PD‐L1/PD‐1 signaling. The cultures were treated with Abs, and luciferase activity was measured by luminescence. Data were analyzed with GraphPad Prism software using a 4‐parameter non‐linear regression to calculate EC_50_ values. Data are presented from three independent experiments.

### 4.8 Ab internalization assay

For the assay, 100,000 Karpas‐299 cells were plated in each well of a 96‐well plate in cell culture media. Plates were chilled on ice for 10 min, and then a chilled solution of an Ab that had been precomplexed with Human Fabfluor‐pH Red Antibody Labeling Reagent (Sartorius, 4722) to provide a final concentration of 25 nM Ab and 75 nM Fabfluor‐pH Red reagent was added to duplicate wells. The 0‐h time point samples were incubated on ice for 10 min followed by washing the cells in PBS to remove unbound Ab and the cells were immediately analyzed by flow cytometry as described below. For the 24‐h time point samples, treated cells were incubated with Abs at 37°C under 5% CO_2_ for a period of 24 h. After treatment, cells were stained using a viability dye (ThermoFisher, 65‐0863‐14) and then analyzed using a Cytoflex Flow cytometer. After gating for viable cells, the resulting fluorescence intensity from the allophycocyanin channel was used for data analysis. Data were analyzed in GraphPad Prism and are presented as the average from four independent experiments.

### 4.9 Flow cytometry

L540 cells (10^6^) were washed in ice‐cold PBS supplemented with 0.5% BSA (Assay Buffer), added to a 96‐well plate, and incubated with a dilution of the indicated Abs for 30 min on ice. Unbound Abs were removed by washing cells three times with Assay Buffer. Bound Abs were detected using an AlexaFluor 488 conjugated rabbit anti‐human IgG F(ab’)2 (Jackson 309‐545‐006) and cells were stained with Fixable Viability Dye eFluor‐780 (Invitrogen 65‐0865). Data were collected using a CytoFLEX‐S flow‐cytometer (Beckman Coulter) using a 488 nm laser with a 525/40‐nm filter to detect bound Ab and a 638 nm laser with a 780/60‐nm filter to detect viable cells. Quantitation analysis was carried out using FlowJo v10.2 Software (FlowJo, LLC) and GraphPad Prism v11.0.0 (GraphPad Prism, LLC).

### 4.10 Protein production and purification for crystallography experiments

Fabs for crystallization were produced in mammalian cells from genes encoding each heavy and light chains cloned into separate vectors with a human N‐terminal trypsinogen‐2 signal peptide. Additional crystallization‐enhancing substitutions were incorporated into the Fab framework to match those of the S1CE2 framework (Bruce, Singer, Blazer, et al., 2024). As per manufacturer's instructions, Expi293 cells were grown to a density of 2 × 10^6^ cells/mL in Expi293 media (Gibco) prior to co‐transfection with a mixture of Fab heavy and light chains using FectoPRO (Sartorius; 101000007). Cells were incubated at 37°C, 8% CO_2_, and 80% humidity with shaking at 125 rpm for 5 days. Fabs were purified from the supernatant media after clarification by centrifugation followed by incubation with rProtein A Sepharose™ beads (Cytiva GE17‐1279‐03) to capture proteins for 16 h at 4°C with shaking at 125 rpm. Beads were collected and bound Fab protein was eluted with IgG elution buffer (Pierce; 21004) and exchanged into 20 mM 4‐(2‐hydroxyethyl)‐1‐piperazineethanesulfonic acid (HEPES) pH 7.5, 100 mM NaCl buffer using 30 kDa molecular weight cut‐off (MWCO) concentrators prior to evaluation of purity by SDS‐PAGE and quantification by spectrophotometric absorbance at 280 nm.

The DNA sequence encoding the human PD‐L1^NTD^ domain (aa 18–132, UniProt Accession Q9NZQ7.1) or CD30^CRD2/3^ domain (aa 66–153, UniProt Accession P28908.1) with an N‐terminal trypsinogen‐2 signal peptide and a C‐terminal thrombin cleavage site followed by a hexa‐histidine tag was cloned into mammalian expression vectors and expressed in Expi293 cells as described above. Prior to transfection, 5 μM kifunensine was added to the cell culture media to inhibit mannosidase I for later deglycosylation by EndoH (New England Biolabs). Protein was isolated from clarified media by incubation with immobilized metal affinity chromatography using Ni Sepharose™ excel resin for 16 h at 4°C with shaking at 125 rpm. Protein was eluted from the beads with 200 mM imidazole in 20 mM HEPES, 100 mM NaCl and exchanged into the same buffer without imidazole using a 30 kDa MWCO concentrator. Protein purity was evaluated by SDS‐PAGE and quantified by spectrophotometric absorbance at 280 nm.

### 4.11 Crystallographic screening

Fab:PD‐L1 and Fab:CD30 complexes were prepared in a 1.1:1 molar ratio and separated by SEC on a Superdex™ 200 increase10/300 GL column equilibrated with 20 mM HEPES, 100 mM NaCl. Fractions containing only complex (as determined by SDS‐PAGE) were combined and concentrated to 5 mg/mL for crystallization screening with commercial conditions from screens including JCSG+ECO™ and PACT Premier™ (Molecular Dimensions), and SaltRX™ and GRAS1™ (Hampton Research) by sitting drop vapor diffusion in 3‐well low volume ARI INTELLI‐PLATE 96‐well plates (ArtRobbins, 102‐0001‐03). Screens were set using an ArtRobbins Gryphon with 0.25 μL complex to 0.25 μL condition drops and 50 μL condition reservoir. Crystallographic statistics and PDB Ids can be found in Table S2.

Crystals used for data collection were produced in conditions and cryoprotected as described below.

The Fab‐P^1a^:PD‐L1 complex crystallized at 5 mg/mL in 20 mM HEPES pH 7.5, 100 mM NaCl in a 1 μL:1 μL ratio with 5.5M sodium formate, 0.1M sodium acetate trihydrate pH 4.6, at a final pH of 5.8. A 200 μm prism crystal was cryoprotected with parabar 10,312 prior to vitrification in liquid nitrogen.

The Fab‐P^2a^:PD‐L1 complex crystallized at 5.5 mg/mL in 20 mM HEPES pH 7.5, 100 mM NaCl in a 1.5 μL:1.5 μL ratio with 0.3M ammonium citrate and 20% w/v polyethylene glycol 3350. A 200 μm rod crystal was cryoprotected with trough solution mixed with 30% v/v glycerol prior to vitrification in liquid nitrogen.

The Fab‐P^3a^:PD‐L1 complex crystallized at 5.5 mg/mL in 100 mM bis‐tris methane pH 6, 100 mM NaCl in a 1 μL:1 μL ratio with 0.1M sodium citrate pH 5.5 and 20% w/v polyethylene glycol 1500. A 300 μm rod crystal was cryoprotected with trough solution mixed with 30% v/v ethylene glycol prior to vitrification in liquid nitrogen.

The Fab‐C^1^:CD30 complex crystallized at 3.3 mg/mL in 20 mM HEPES pH 7.5, 100 mM NaCl in a 1 μL:1 μL ratio with 0.1M bis‐tris methane pH 5.5 and 20% w/v polyethylene glycol 3350. A 300 μm plate crystal was cryoprotected with trough solution mixed with 30% v/v ethylene glycol prior to vitrification in liquid nitrogen.

### 4.12 Diffraction data collection

All datasets were collected at Brookhaven National Laboratory National Synchrotron Light Source II on beamline frontier microfocusing macromolecular crystallography beamline (Schneider et al., 2021) 17‐ID‐2 (P^1a^:PD‐L1) or automated macromolecular crystallography beamline (Schneider et al., 2022) 17‐ID‐1 (remaining datasets). Data were either processed at the beamline using AUTOPROC (Vonrhein et al., 2011), or separately with DIALS/XIA2 within CCP4 (Winter et al., 2013). Data were merged and scaled using AIMLESS (Evans & Murshudov, 2013) either within the AUTOPROC pipeline or from CCP4. Data resolution was based on an overall signal to noise ratio (*I*/*σ*) of >2 following data merging using AIMLESS (Evans & Murshudov, 2013). All structures were solved by PHASER (McCoy et al., 2007) in the Phenix crystallography suite, followed by iterative cycles of refinement using phenix.refine (Zwart et al., 2008) and manual refinement of the resulting model and electron density map using the molecular graphics package COOT (Emsley & Cowtan, 2004). Translation‐liberation‐screw (TLS) (Urzhumtsev et al., 2013) parameterization with group B‐factor refinement was also used. Ramachandran restraints were also used during refinement of all structures. Using the structure of PD‐L1^NTD^ (PDB code 5C3T) and the S1CE2 Fab framework structure (PDB code 8VUC), a solution for molecular replacement for each complex was determined. This required using a search procedure that first found the Fab, then searching for PD‐L1^NTD^ or manually building CD30^CRD2/3^ into the electron density.

## AUTHOR CONTRIBUTIONS

**Evan Mallette:** Conceptualization; investigation; writing – original draft; writing – review and editing; visualization; formal analysis; data curation; methodology. **Levi L. Blazer:** Conceptualization; investigation; writing – original draft; writing – review and editing; visualization; methodology; formal analysis; data curation; supervision. **Craig A. Hokanson:** Investigation; methodology; validation; formal analysis; data curation. **Chao Chen:** Investigation; methodology; validation; formal analysis; data curation. **Julia Garcia Perez:** Investigation; methodology; validation; formal analysis; data curation. **Alevtina Pavlenco:** Investigation; methodology; validation; formal analysis; data curation. **Lynda Ploder:** Investigation; methodology; validation; formal analysis; data curation. **Alexander U. Singer:** Conceptualization; investigation; validation; visualization; methodology; writing – review and editing; formal analysis. **Michael D. L. Suits:** Supervision; project administration; resources; funding acquisition. **Sunil Bhakta:** Conceptualization; investigation; validation; methodology; formal analysis; data curation; writing – review and editing. **Jagath R. Junutula:** Conceptualization; writing – review and editing; formal analysis; project administration; resources; supervision; data curation; methodology. **Jarrett J. Adams:** Conceptualization; investigation; methodology; validation; writing – review and editing; formal analysis; data curation; supervision; resources; project administration. **Sachdev S. Sidhu:** Conceptualization; investigation; funding acquisition; writing – original draft; writing – review and editing; methodology; formal analysis; project administration; supervision; data curation; visualization.

## CONFLICT OF INTEREST STATEMENT

Abs P^2a^, P^3a^, P^4a^, and C^1^ are licensed to and patented by Aarvik Therapeutics. Sachdev S. Sidhu, Levi L. Blazer, and Jarrett J. Adams are advisors and shareholders of Aarvik Therapeutics. Sunil Bhakta and Jagath R. Junutula are employees and shareholders of Aarvik Therapeutics. Sachdev S. Sidhu and Jarrett J. Adams are employees of Simisco Biosciences, and Sachdev S. Sidhu is a shareholder of Simisco Biosciences.

## Supporting information


**Data S1.** Supporting Information.

## Data Availability

The data that support the findings of this study are available from the corresponding author upon reasonable request.
