# Supplementary material for: Strategy for modular assembly of tetravalent, multispecific antibodies
Source: Protein Sci. 2026 Sep 18;35(10):e70797. doi: 10.1002/pro.70797 (PMC13587143; doi:10.1002/pro.70797)
Supplement: Supplementary file 1 [file PRO-35-e70797-s001.docx]

**Supplementary Information for**

**Strategy for Modular Assembly of Tetravalent, Multispecific Antibodies**

Evan Mallette^1,3^*, Levi L. Blazer^1,4^*, Craig A. Hokanson^2^, Chao Chen^1^, Julia Garcia Perez^1^, Alevtina Pavlenco^1^, Lynda Ploder^1^, Alexander U. Singer^1^, Michael D. L. Suits^3^, Sunil Bhakta^4^, Jagath Reddy Junutula^4^, Jarrett J. Adams^1,2,4^, Sachdev S. Sidhu^1,2,4^^

**Table S1. Surface area on Fabs buried upon antigen binding.** Total surface area buried on each Fab upon complexation with antigen was determined by PISA. All values are given in Å^2^ units. Headings are as follows: Fab (Fab name), Heavy Chain (heavy chain buried surface area), Light Chain (light chain buried surface area), Total (total buried surface area). Also shown are buried surface areas contributed by each of the six CDRs, the light chain framework (LC-FW) and heavy chain framework (HC-FW).

**Figure S1. SDS-PAGE analysis of IgG and multivalent Abs. (a)** Reducing and non-reducing PAGE analysis of IgGs P^2a^, P^3a^, and P^4a^. **(b)** Reducing and non-reducing PAGE analysis of IgGs BRNT and C^1^. **(c)** Reducing and non-reducing PAGE analysis of AV-P087, 88, 89, and 90.

**(a)**

**(b)**

**(c)**

CD30^CRD 2/3^

AF-TNFR2^69-152^

CD30^CRD 2/3^

AF-CD30^78-163^

TNF^9-158^

AF-CD30L^105-215^

**Figure S2. Alignment of CD30 and TNF structures with AlphaFold models.** PyMol alignment of the structure of CD30^CRD2/3^ (cyan) with **(a)** the AlphaFold model of TNFR2 (tan) and with **(b)** the AlphaFold model of CD30^CRD2/3^ (orange). **(c)** PyMol alignment of TNF (magenta, PDB 3ALQ chain A) with AlphaFold model of CD30L (grey).

**Figure S3. Contacts between CDR-H3 and light-chain CDRs.** Contact residues (<4 Å) between CDR-H3 (red) and CDRs L1 (blue), L2 (azure blue), and L3 (lavender) are shown for Fabs **(a)** ER-2a, **(b)** ER-3c, **(c)** C^1^, **(d)** P^2a^, **(e)** P^3a^, and **(f)** P^4a^. The main chains are shown as tubes and contact side chains are shown as sticks. Hydrogen bonds are shown as dashed yellow lines.

**Table S2.** Crystallography Statistics.

|  | **PD-L1/ Fab^S1CE2^_P2a** | **PD-L1/ Fab^S1CE2^_P3a** | **PD-L1/ Fab^S1CE2^_P4a** | **CD30/**  **Fab^S1CE2^_C1** |
| --- | --- | --- | --- | --- |
| **PDB Code** | 13FL | 13DS | 13DT | 13HA |
| **Beamline** | NSLSII-17-ID-2 | NSLSII-17-ID-1 | NSLSII-17-ID-1 | NSLSII-17-ID-1 |
| **Crystals** | Native | Native | Native | Native |
| **Unit Cell parameters** |  |  |  |  |
| **Space group** | C222_1_ | P2_1_2_1_2_1_ | P2_1_ | P3_1_2 |
| **a, b, c (Å)** | 66.4, 239.7, 104.3 | 61.4, 71.9, 190.5 | 69.6, 72.3, 72.5 | 72.2, 72.2, 220.0 |
| **𝜶, β, 𝜸 (°)** | 90, 90, 90 | 90, 90, 90 | 90, 117.5, 90 | 90, 90, 120 |
| **Resolution (Å)** | 40.41-1.97  (2.28-1.97)* | 33.62-1.93  (2.03-1.93) | 34.80-1.64  (1.73-1.64) | 220.04-2.0  (2.03-2.0) |
| **Unique Reflections** | 27786 (1389) | 63557 (9071) | 77525 (11254) | 46203 (2246) |
| **R_pim_** | 0.122 (0.922) | 0.048 (0.828) | 0.062 (1.007) | 0.053 (0.808) |
| **Overall I /𝜎I** | 8.3 (1.4) | 8.0 (1.1) | 6.6 (0.7) | 12.0 (0.8) |
| **Completeness (%)** | 90.9 (74.2) | 98.6 (97.8) | 100 (100) | 100 (99.0) |
| **Redundancy** | 8.8 (8.8) | 8.7 (9.0) | 7.0 (6.6) | 20 (18.6) |
| **CC half** | 0.995 (0.213) | 0.998 (0.370) | 0.996 (0.461) | 0.998 (0.394) |
| **Wilson *B* Factor** | 28.6 | 30 | 17.8 | 11.7 |
| **Refinement** |  |  |  |  |
| **Resolution (Å)** | 40.41-2.22 | 33.62-1.93 | 34.80-1.64 | 47.56-2.0 |
| **No. reflections** | 26827 (1324)** | 53788 (2765) | 64582(3200) | 46016 (2366) |
| **R_work_ / R_free_ (%)** | 19.9/23.9 | 17.4/20.7 | 17.4/20.7 | 18.9/22.2 |
| **No. atoms** |  |  |  |  |
| **Protein** | 4201 | 4174 | 4304 | 3918 |
| **Water** | 94 | 497 | 541 | 537 |
| **Other** | 21 | 84 | 147 | 62 |
| **B-factors** |  |  |  |  |
| **Overall** | 33.3 | 35.6 | 22.9 | 44 |
| **Protein** | 33.3 | 34.6 | 21.5 | 42.8 |
| **Water** | 28.9 | 40.9 | 30.7 | 51.8 |
| **Ligand/ion** | 56.8 | 53.4 | 34.7 | 49.5 |
| **R.m.s. deviations** |  |  |  |  |
| **Bond lengths (Å)** | 0.009 | 0.007 | 0.007 | 0.008 |
| **Bond angles (°)** | 1.06 | 0.91 | 0.88 | 0.94 |
| **Ramachandran** |  |  |  |  |
| **Favoured** | 96.1 | 97 | 97 | 97.3 |
| **Allowed** | 3.5 | 2.8 | 2.8 | 2.7 |
| **Outliers** | 0.4 | 0.2 | 0.2 | 0 |

*Value in parentheses represent highest-resolution shell.
**Value in parenthesis represents the number of reflections in the test set.
